# Elucidating chiral myosin–induced actin dynamics: From single-filament behavior to collective structures

**DOI:** 10.1073/pnas.2508686123

**Published:** 2026-01-28

**Authors:** Takeshi Haraguchi, Kohei Yoshimura, Yasuhiro Inoue, Takuma Imi, Koyo Hasegawa, Taisei Nagai, Hideki Furusawa, Toshifumi Mori, Kenji Matsuno, Kohji Ito

**Affiliations:** ^a^Department of Biology, Graduate School of Science, Chiba University, Chiba 263-8522, Japan; ^b^Center of Quantum Life Science for Structural Therapeutics, Chiba University, Chiba 263-8522, Japan; ^c^Department of Micro Engineering, Kyoto University, Kyoto 615-8540, Japan; ^d^Department of Applied Molecular Chemistry, Institute for Materials Chemistry and Engineering, Kyushu University, Fukuoka 816-8580, Japan; ^e^Department of Interdisciplinary Engineering Sciences, Interdisciplinary Graduate School of Engineering Sciences, Kyushu University, Fukuoka 816-8580, Japan; ^f^Department of Biological Sciences, Graduate School of Science, Osaka University, Osaka 560-0043, Japan; ^g^Molecular Chirality Research Center, Chiba University, Chiba 263-8522, Japan; ^h^Plant Molecular Science Center, Chiba University, Chiba 263-8522, Japan

**Keywords:** myosin, actin, collective motion, self-assembly, chirality

## Abstract

The myosin superfamily encompasses over 70 classes, each with multiple subclasses, and exhibits substantial diversity in properties such as velocity, ATPase activity, duty ratio, and directionality. This functional diversity enables the specialized roles of each myosin in various organisms, organs, and cell types. Beyond these well-characterized parameters, a newly recognized property has recently come into focus: Certain myosins drive actin filaments along chiral curved trajectories. However, this newly identified property remains largely unexplored. Here, we investigated this chiral motion in vitro using *Chara corallina* myosin XI (*Cc*XI), which drives fast clockwise (CW) movement of actin filaments. This chiral motion arises from asymmetric displacement at the filament’s leading tip, and its curvature depends on the myosin density. Surprisingly, at elevated actin concentrations, filaments exhibiting chiral curved motion undergo collective dynamics, spontaneously forming a ring-shaped structure—termed the actin chiral ring (ACR)—that exhibits persistent CW rotation. ACRs display remarkable stability, continuing to rotate at their formation site until ATP is depleted, while maintaining their structure even after rotation ceases. This stability has not been reported among reported collective motions of cytoskeletal proteins driven by various motors. Our findings demonstrate that myosins with chiral activity can autonomously organize actin filaments into stable, chiral structures through collective motion, providing insights into actin self-organization by unconventional myosins. This paradigm offers a mechanistic basis for how motor-driven molecular asymmetry can give rise to coherent structural chirality at the cellular scale—an essential step in the emergence of cell chirality and asymmetry during development.

## Significance

Myosins are motor proteins that move along actin filaments and underpin various intracellular functions. Recently, some myosins have been found to drive actin filaments along chiral curved paths, but the mechanisms and significance of this behavior remain largely unexplored. Here, we investigated this activity and found that it not only drives chiral curved motion of single actin filaments but also organizes them into stable, unidirectionally rotating ring structures through collective motion. These rings, termed actin chiral rings (ACRs), spontaneously emerge at high actin concentrations. Our findings uncover a organizing principle of actin self-assembly driven by myosin with chiral activity and provide a framework for understanding how cytoskeletal chirality is established.

Phylogenetic analyses of myosin motor domain (MD) sequences have revealed remarkable diversity within the myosin superfamily. To date, at least 79 distinct classes have been identified (1). These myosin classes and subclasses exhibit broad functional diversity in properties such as actin sliding velocity, actin-activated ATPase activity, duty ratio (the fraction of ATPase cycles during which the MD remains strongly bound to actin), and directionality of actin filament movement (2). Recently, a novel property has been reported, namely that rat myosin IC and *Drosophila* myosin ID drive actin filaments in a CW trajectory from the perspective of the objective lens (myosin side), resulting in chiral curved motions (3, 4). This chiral activity is particularly noteworthy, as the MD of *Drosophila* myosin ID plays a critical role in establishing left–right (L–R) asymmetry at both the cellular and organ levels (4–7). However, the mechanistic link between the chiral activity of myosin ID and the emergence of cellular or organismal chirality remains poorly understood.

Motility characteristics of myosin isoforms—such as sliding velocity and directionality—have been extensively analyzed using in vitro motility assays. In this method, myosin molecules are randomly immobilized onto a coverslip, and fluorescently labeled actin filaments are introduced along with ATP to visualize their movements (8). Although the filaments move in various directions across the surface, their velocity and directionality remain consistent with those observed for the corresponding myosin isoforms in vivo. Therefore, in vitro motility assays serve as a reliable method for quantifying the velocities and directionality of myosin-driven actin movements. These assays typically use fluorescent actin at a concentration of 10 nM (9), which is approximately 1/10,000 of the intracellular actin concentration, as the F-actin concentration in animal cells is estimated to be ~100 µM (10). Such low concentrations are employed to allow for the clear observation of individual actin filament trajectories.

Recently, a modified in vitro motility assay has been developed to enable the tracking of individual actin filaments under high actin concentrations. This improvement was achieved by supplementing the assay buffer with nonfluorescent actin at elevated levels, together with a low concentration of fluorescently labeled actin. Remarkably, under these dense actin conditions, actin filaments driven by skeletal muscle myosin II (SkII) no longer exhibited random motions. Instead, they displayed collective behaviors characterized by coordinated movement; moving actin filaments are aligned along their longitudinal axes (11–19). Moreover, under certain conditions, not only the filament axes but also their polarities become aligned, resulting in unidirectional motion of the filaments (12–17).

To date, most in vitro studies on actin filament-based collective motion have employed SkII, also known as conventional myosin (11–19). However, within muscle sarcomeres—where SkII is endogenously localized—both myosin and actin are fixed, and collective motion does not occur. In contrast, intracellular actin movements in nonmuscle animal cells and plant cells are primarily driven by unconventional myosins. These movements often exhibit ordered filament orientation, suggesting that unconventional myosins may contribute to intracellular actin alignment through collective motion. Given that unconventional myosins differ in their motile properties from SkII, their collective dynamics are also likely to be distinct.

Collective motion of active matter is strongly governed by the geometry and motile properties of its components. For rod-shaped active matter, such as those composed of elongated filaments, collective motion is predominantly driven by lateral interactions, resulting in alignment along the longitudinal axes. This phenomenon has been observed in the collective motion of actin filaments driven by SkII, where filaments behave as linear rods and exhibit motion aligned with their long axes, as described previously (11–19). Recently, increasing attention has been directed toward the collective motion of chiral active matter, in which the components either possess intrinsic chirality or exhibit chiral motion patterns (20, 21). FtsZ, a bacterial homolog of the eukaryotic protein tubulin, polymerizes into filaments. When these filaments are anchored to a lipid surface via FtsA, they exhibit a treadmill-like motion and adopt a chiral shape. At high concentration, these filaments self-organize into rotating chiral rings, a process proposed to arise from lateral interactions between filaments with chiral geometry (22, 23).

In this study, we demonstrate that the MD of *Chara corallina* myosin XI (*Cc*XI MD) induces CW actin movements when viewed from the objective lens side (i.e., myosin side), similar to that reported for rat myosin IC (3) and *Drosophila* myosin ID (*Dm* ID) (4). We also show that chiral motion driven by *Cc*XI MD induces the self-organization of actin filaments into stable, unidirectionally rotating structures, which we term actin chiral rings (ACRs). This study builds upon our earlier preprint published in March 2025, in which we first described ACR formation (24). Therefore, the current work represents a peer-reviewed report of ACRs, establishing a mechanism of cytoskeletal self-organization driven by unconventional myosin with chiral activity. Subsequently, we demonstrated that *Dm* ID, previously shown to induce chiral motion of individual actin filaments, can also form ACRs under high actin concentrations, as reported in our preprint published in May 2025 (25). However, ACR formation driven by *Dm* ID in the in vitro motility assays requires a prolonged timescale (approximately 60 to 90 min), during which continuous tracking of actin filaments is not feasible, thereby precluding detailed temporal analysis of the ACR formation process. In contrast, the substantially higher motility speed of *Cc*XI MD enables real-time observation of the entire ACR formation process, allowing us to uniquely elucidate the temporal sequence and mechanistic steps underlying ACR emergence in the current study. Taken together, ACRs driven by myosins with chiral activity provide insights into the mechanisms of actin filament self-organization and broaden our understanding of the functional diversity of unconventional myosins in cytoskeletal dynamics.

## Results

### Chiral Motion of Actin Filaments by *Cc*XI.

**Fig. 1.**
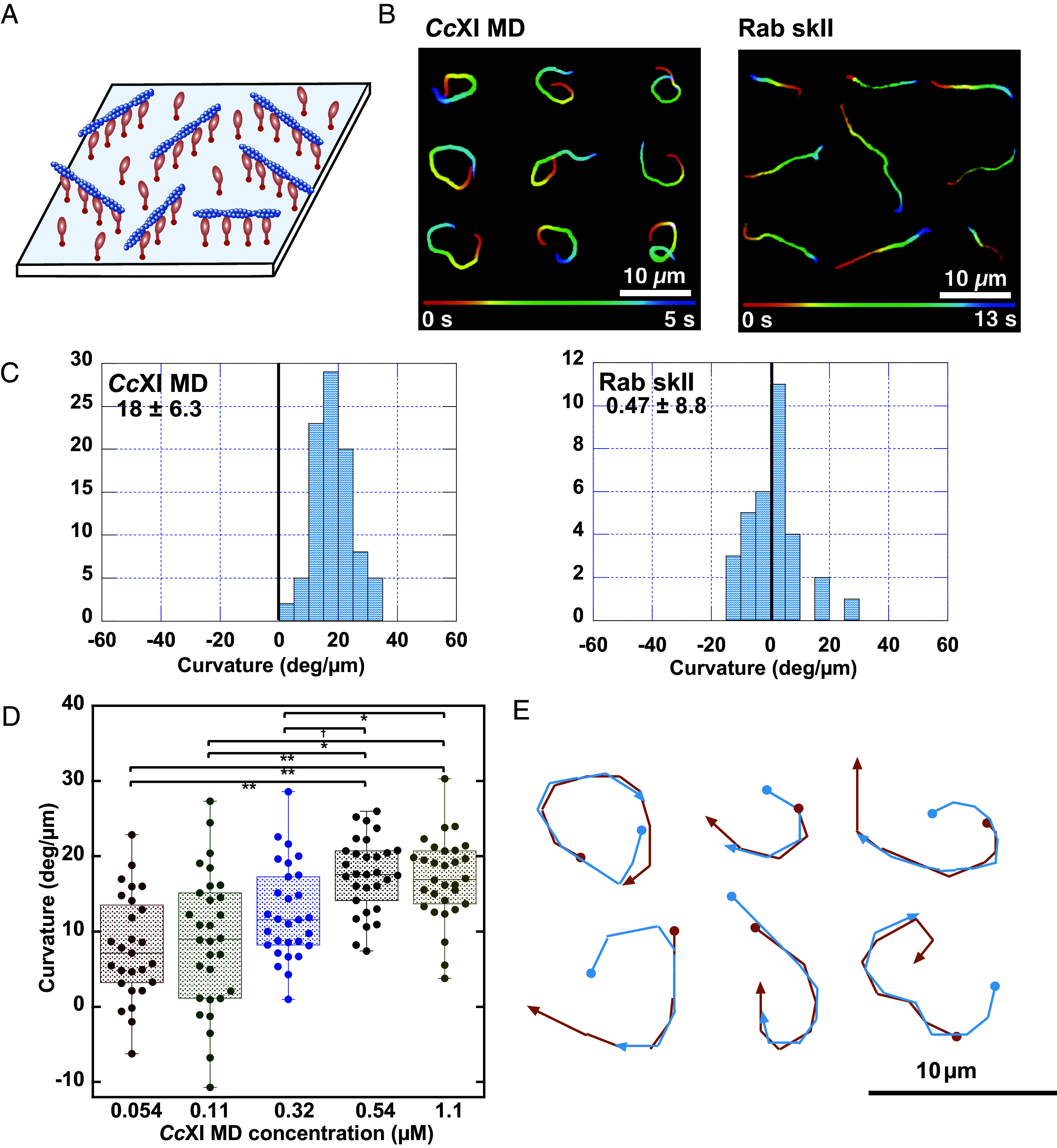
Chiral curved motion of actin filaments driven by *Cc*XI in the standard in vitro motility assay (*A*) Schematic of the standard in vitro motility assay. (*B*) Representative trajectories of actin filaments driven by *Cc*XI MD and rabbit skeletal muscle myosin II (Rab SkII). Trajectories indicate filament motion over 5 s (*Cc*XI) and 13 s (Rab SkII); colors represent time progression. *Cc*XI MD filaments moved CW when viewed from the objective lens side (Movie S1). (*C*) Histogram of filament curvatures with *Cc*XI MD and Rab SkII. Positive values indicate CW motion. For *Cc*XI MD, curvature measurements were obtained from three independent protein preparations (*N* = 3), yielding a total of 112 filaments with an overall mean curvature of 18 ± 6.3 deg/µm. Values for each preparation were as follows: 17 ± 5.5 deg/µm (*n* = 30 filaments), 21 ± 6.3 deg/µm (*n* = 50 filaments), and 16 ± 6.1 deg/µm (*n* = 32 filaments). (*D*) Box plot of filament curvature at various concentrations of *Cc*XI MD. Because the curvature–concentration relationship varies between independent protein preparations, all measurements in this panel were performed using a single protein preparation (*N* = 1) to ensure internally comparable concentration dependence; multiple filaments were analyzed at each concentration (*n* values are indicated in the plot). Statistical analysis was performed using one-way ANOVA with Tukey’s HSD test; significant differences are indicated (*SI Appendix, Supplementary Materials and Methods*). (*E*) Overlay of leading (red) and trailing (blue) filament ends shows curvature originates at the leading tip.

In the standard in vitro motility assay (Fig. 1*A*), we found that *Cc*XI MD, a plant myosin XI previously highlighted as a fast myosin (26–28), drove actin filaments in a CW direction when viewed from the objective lens side (myosin side) (Fig. 1*B*: *Cc*XI MD and Movie S1). Similar CW movements have been reported for actin filaments driven by mouse myosin IC (3) and *Drosophila* myosin ID (4). The curvature of actin movements driven by *Cc*XI MD was 18 ± 6.3 deg/µm (Fig. 1*C*: *Cc*XI MD), which exceeds the values reported for mouse myosin IC and *Drosophila* myosin ID using a similar assay (standard in vitro motility assay on glass surfaces) (3, 4). In contrast, actin filaments driven by rabbit skeletal muscle myosin II (SkII)—a widely used reference myosin—followed nearly straight trajectories, with an average curvature of 0.47 ± 8.8 deg/µm (Fig. 1 *B* and *C*: Rab SkII).

To determine whether this chiral activity is a common feature among class XI myosins, we measured the curvature of the actin trajectories using *Cb*XI-3 (*Chara braunii* myosin XI-3) MD, a paralog of *Cc*XI that shares high sequence similarity (98%) and identity (88%) (29). Despite this similarity, the curvature induced by *Cb*XI-3 MD (3.1 ± 8.7 deg/µm) was significantly smaller than that induced by *Cc*XI MD. These results suggest that the curvature of the trajectory is determined by subtle variations in the myosin amino acid sequence, even among class XI myosins. This finding is consistent with previous reports indicating that only a subset of class I myosins generate curved actin trajectories whereas most do not (3, 4).

### Mechanistic Features of Chiral Motion.

To investigate the dependence of actin filament curvature on myosin density, we performed a series of in vitro motility assays. In these assays, *Cc*XI MD was immobilized on the coverslip surface in chambers with a spacer thickness of 0.24 mm, and the concentration of myosin introduced into the chamber was systematically varied. As shown in Fig. 1*D*, the curvature of actin filaments increased with rising concentrations of *Cc*XI MD. Statistical analysis (*SI Appendix, Supplementary Materials and Methods*) revealed significant differences in curvature between 0.54 µM and lower concentrations (0.054 and 0.11 µM), as well as between 1.1 µM and all lower concentrations. The difference between 0.32 µM and 0.54 µM was marginally significant (*P* = 0.052), suggesting a potential threshold effect near 0.5 µM. At concentrations ≥0.54 µM, curvature values appeared to plateau, indicating saturation of the myosin-binding sites on the surface. At this concentration, the surface myosin density was estimated to be approximately 3.6 molecules per 10 × 10 nm^2^ area. Given that the diameter of the myosin XI MD is ~4 nm (29), this density likely corresponds to near-saturation of the coverslip surface. These results indicate that actin filament curvature increases in proportion to surface myosin density until saturation is reached. The potential mechanism underlying this density-dependent increase in curvature is further discussed in the *Discussion* and *SI Appendix*, Fig. S1.

To elucidate the mechanism underlying this chiral curved motion, we tracked both the leading and trailing ends of individual actin filaments. We found that both ends followed the same trajectory (Fig. 1*E*), indicating that the curved motion is primarily generated by asymmetric displacement at the filament’s leading tip, while the remainder of the filament passively follows the path without actively contributing to the curvature. This observation suggests that the leading tip of the filament is uniquely permissive to curvature, potentially due to localized flexibility, as further discussed in the *Discussion* and *SI Appendix*, Fig. S2.

### Collective Actin Movements Leading to ACRs.

Conventional in vitro motility assays typically use ~10 nM fluorescent F-actin (9), far below the intracellular concentration of ~100 µM in animal cells (10) because high levels of fluorescently labeled actin obscure individual filaments. This limitation has been overcome by mixing a small fraction of labeled actin with a large excess of unlabeled filaments, enabling motility assays under dense conditions. Using skeletal muscle myosin II (SkII), two major protocols have been established to examine actin dynamics under dense actin conditions, namely Molloy and Bausch conditions. Notably, under such dense conditions, collective actin movements emerge that are not readily observed in conventional low-density in vitro motility assays. In the Molloy condition, actin filaments introduced together with ATP exhibit nematic streaming characterized by bidirectional motion, indicative of orientational order without global polarity sorting (11). In contrast, under the Bausch condition, actin filaments are first allowed to bind to surface-immobilized myosin in the absence of ATP, after which unbound filaments are removed by washing. This procedure eliminates excess filaments in solution, thereby restricting motility to surface-bound filaments. Upon subsequent ATP perfusion, pronounced polar streaming emerges in a density-dependent manner, a behavior that is not prominent under the Molloy condition (12–14).

Building on these established frameworks, we investigated collective actin dynamics driven by *Cc*XI MD, focusing on the conditions under which chiral collective motion emerges. For clarity, we classify actin density regimes based on the measured surface filament density: ~5 to 20 filaments/µm^2^ as a moderately high actin density regime, ~20 to 50 filaments/µm^2^ as a high actin density regime, and ≥~60 filaments/µm^2^ as an ultra–high actin density regime, corresponding to near-saturated surface coverage under our experimental conditions.

### In Vitro Motility Assay of *Cc*XI MD under the Bausch Condition.

Under the Bausch condition, unlabeled actin filaments (2.4, 7.1, or 24 µM), supplemented with a small fraction of fluorescently labeled filaments, were introduced into chambers coated with *Cc*XI MD under ATP-free conditions. After allowing filaments to bind to surface-immobilized myosin, unbound filaments were removed by washing with ATP-free buffer. Actin motility was then initiated by infusion of ATP-containing buffer (*SI Appendix, Supplementary Materials and Methods, In Vitro Motility Assay under the Bausch Condition*).

**Fig. 2.**
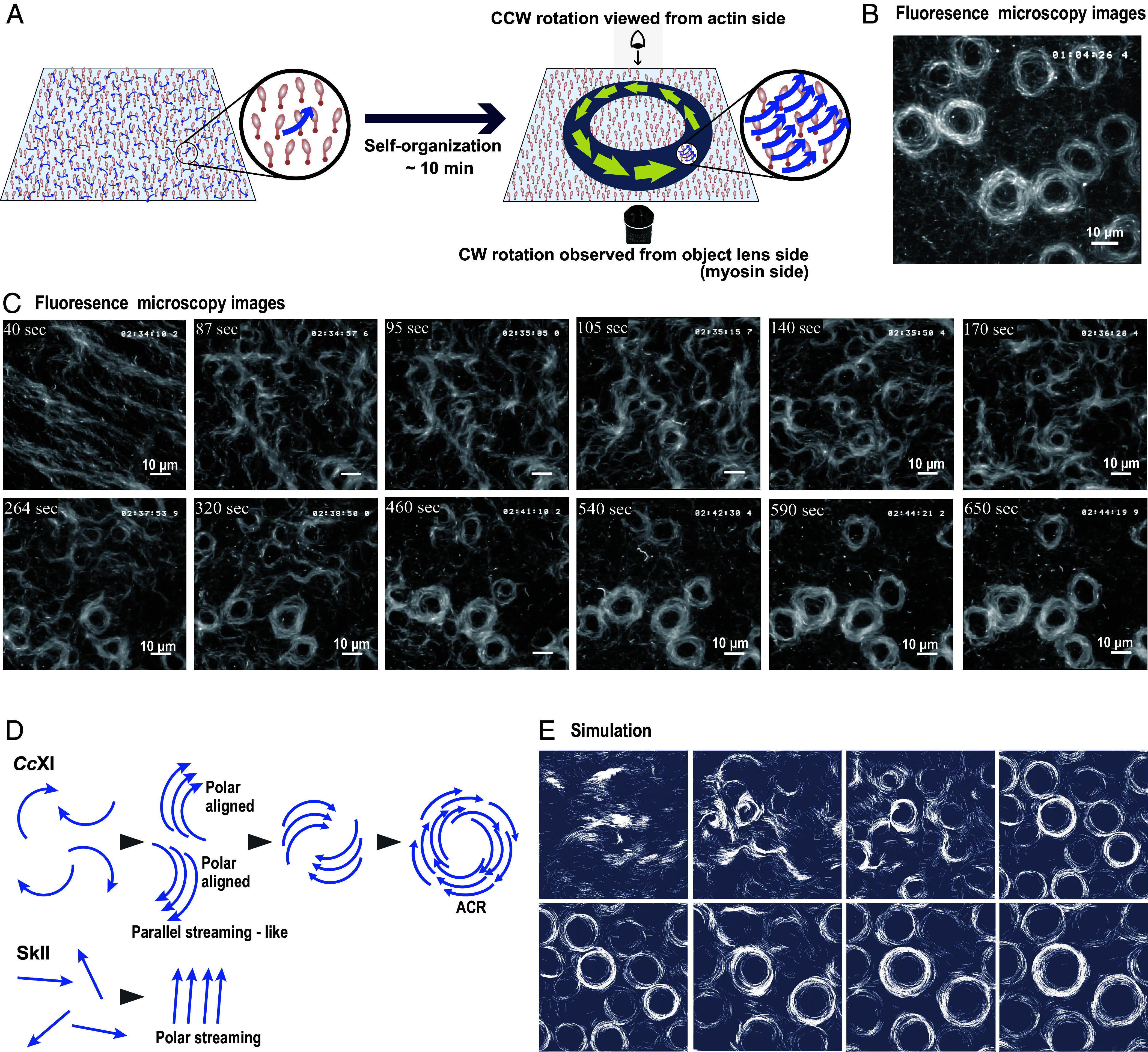
Autonomous formation of ACRs through the collective motion of chiral curved filaments (*A*) Schematic of ACR formation under elevated actin density. (*B*) Fluorescence microscopy image of a fully formed ACR rotating CW at its original site (Movie S2). (Scale bar, 10 µm.) (*C*) Time-lapse images of ACR formation: initial stream-like filament flow after ATP addition, followed by coalescence into nascent rings and maturation into stable ACRs (Movie S7). (*D*) Schematic comparing collective motion: *Cc*XI-driven curved filaments form rotating rings, whereas SkII-driven straight filaments form polar streams. (*E*) Simulation of chiral curved filaments shows spontaneous transition from streaming to rotating ring structures, consistent with experimental observations (Movie S7). All experiments shown in this figure were conducted under the *Bausch condition* (moderately high actin density).

Under the Bausch condition with an initial actin concentration of 2.4 µM, collective filament motion gradually self-organized into stable ACRs approximately 10 min after ATP addition (Fig. 2 *A* and *B* and Movie S2). These ACRs rotated predominantly in the clockwise (CW) direction when viewed from the objective lens side (myosin side) (Fig. 2 *A* and *B* and Movie S2), consistent with the trajectories observed for individual actin filaments at lower actin concentrations. ACRs formed under both low-salt (25 mM KCl, Movie S2) and high-salt (150 mM KCl, Movie S3) conditions, demonstrating that ring formation is not restricted to a narrow ionic regime.

Importantly, under this condition, the surface density of actin filaments reached 15 ± 4.1 filaments/µm^2^ (*SI Appendix,* Fig. S3), placing the system in a moderately high actin density regime, which falls within the threshold range (5 to 20 filaments/µm^2^). This density corresponds to the regime previously reported to support the emergence of polar clusters of actin filaments in the SkII-driven system (12). This quantitative correspondence suggests that ACR formation is governed by a common density-dependent principle underlying collective actin dynamics.

At an initial actin concentration of 7.1 µM under the Bausch condition, surface density increased to 41 ± 3.1 filaments/µm^2^ (*SI Appendix*, Fig. S3), corresponding to a high actin density regime. Following ATP addition, filaments initially exhibited nematic streaming aligned with the direction of solution inflow, which subsequently transitioned into polar streaming. This sequence of nematic-to-polar streaming is consistent with collective behaviors previously reported for skeletal muscle myosin II (SkII) under the Bausch condition at comparable filament densities, where polar clusters and polar streaming constitute the dominant collective states. Notably, SkII-driven systems have also been reported to exhibit polar streaming at comparable filament densities in this high-density regime (12). Although ACRs eventually formed, their appearance was delayed and their frequency was reduced (*SI Appendix,* Fig. S4 and Movie S4) compared with the moderately high actin density condition.

At an initial actin concentration of 24 µM under the Bausch condition, surface density reached 75 ± 10 filaments/µm^2^ (*SI Appendix,* Fig. S3), corresponding to an ultra–high actin density regime. Assuming an average filament length of ~2 µm, this density would correspond to a near-saturated surface coverage. Immediately after ATP addition, actin filaments exhibited nematic streaming aligned with the direction of solution inflow, followed by the emergence of persistent polar streaming. At this density, collective dynamics closely resembled those reported for SkII-driven systems under the Bausch condition, in which polar streaming remains the dominant long-lived state (12). In contrast, in the *Cc*XI-driven system, ACRs occasionally appeared but were spatially heterogeneous and infrequent. In many regions, persistent polar streaming remained the prevailing collective mode even after ACRs had formed elsewhere within the same chamber (*SI Appendix,* Fig. S5 and Movie S5). These observations indicate that at ultra–high actin densities, strong alignment and crowding kinetically suppress the global transition into closed ring structures.

Together, these results reveal a density-dependent hierarchy in *Cc*XI-driven collective actin dynamics under the Bausch condition. Robust ACR formation occurred within a moderately high-density regime (~15 filaments/µm^2^), where filament interactions were frequent but not constrained by crowding. At higher densities (~40 filaments/µm^2^), extended streaming states were stabilized, delaying ACR formation. At ultra–high densities approaching surface saturation (~75 filaments/µm^2^), strong alignment and crowding kinetically suppressed global closure into rotating rings. The actin densities, experimental conditions, and collective behaviors observed under the Bausch condition across the three density regimes are systematically summarized in *SI Appendix*, Table S1.

### In Vitro Motility Assay of *CcXI* MD under the Molloy Condition.

Under the Molloy condition, a solution containing 24 µM unlabeled actin filaments together with ATP was directly perfused into chambers coated with *Cc*XI MD (*SI Appendix, Supplementary Materials and Methods, In Vitro Motility Assay under the Molloy Condition*). Immediately after perfusion, actin filaments became aligned predominantly parallel to the direction of solution inflow, consistent with hydrodynamic effects during chamber loading. As reported previously for skeletal muscle myosin II under the same condition (11), filament motion was bidirectional rather than polarity sorted, indicative of nematic streaming. Over an observation period of ~30 min, nematic streaming persisted, with the preferred orientation varying across regions, but no higher-order structures such as ACRs were observed (*SI Appendix*, Fig. S6 and Movie S6).

Under the Molloy condition with 24 µM actin, the surface density of actin filaments reached 71 ± 8.5 filaments/µm^2^ (*SI Appendix*, Fig. S3), corresponding to near-saturated surface coverage and comparable to that under the Bausch condition at the same actin concentration. Actin filaments persistently exhibited nematic streaming and did not transition to polar waves or ACR formation (*SI Appendix*, Fig. S6 and Movie S6), in clear contrast to the Bausch condition at an initial actin concentration of 24 µM, in which nematic streaming reproducibly transformed into polar streaming and subsequent ACR formation despite similar surface actin densities (*SI Appendix*, Fig. S5 and Movie S5). This difference is likely due to the large excess of actin filaments remaining in the bulk solution under the Molloy condition, which stabilizes nematic order and suppresses polarity sorting at the surface. The collective behaviors observed under the Bausch and Molloy conditions, together with the corresponding actin densities and ACR formation outcomes, are systematically summarized in *SI Appendix*, Table S1.

### The Process of ACR Formation.

Because the Bausch condition using 2.4 µM unlabeled actin reproducibly and robustly yielded ACR formation, we used this condition as a reference setting to analyze the formation process with high temporal resolution. We therefore next examined the time-resolved evolution of actin organization under this condition (Fig. 2*C* and Movie S7). Immediately after ATP addition, as observed under both the Molloy condition and the Bausch condition at higher initial actin concentrations (7.1 µM and 24 µM), actin filaments formed nematic streams aligned along the direction of solution inflow. However, under the Bausch condition with 2.4 µM actin, this nematic streaming did not transition into the polar streaming phase observed at higher actin concentrations. Instead, nematic order rapidly gave way to subsets of curved filaments, which gradually coalesced into nascent ACRs. These immature ACRs began to appear at ~90 s and increased in number until ~300 s, although some disappeared during this period. The surviving nascent ACRs continued to expand in both diameter and width, reaching a more mature but still dynamic stage by ~400 s. By ~600 s, they stabilized into fully developed ACRs that exhibited persistent CW rotation while remaining fixed in position (Fig. 2*C* and Movie S7).

To gain mechanistic insight into how such ring structures could arise from collective filament motion, we compared our results to previously characterized systems driven by SkII. In those systems, polar alignment of filaments has been attributed to orientation changes upon collisions between filaments, resulting in polar streaming (12–14). A similar alignment mechanism could potentially operate in systems driven by *Cc*XI, despite its generation of chiral curved trajectories. If the curved filaments also align their movement direction upon collision, ring formation may be a natural outcome (Fig. 2*D*).

To explore this possibility, we performed in silico simulations to test whether the collective behavior of filaments could reproduce ring formation under different motility assumptions. First, we modeled filaments with straight trajectories that aligned upon collision. As expected, the filaments gradually oriented along their long axes; however, the resulting structure was nematic, with filaments aligned along their long axes but moving in both directions (Movie S8). This suggests that additional conditions—such as polarity-biased alignment, as proposed in the polar streaming model by Bausch et al. (13, 14)—may be necessary to achieve polar ordering in systems of straight-moving filaments.

In contrast, when we modeled filaments with intrinsic chiral curved motion using the same alignment rules, spontaneous formation of ring-like structures emerged (Fig. 2*E* and Movie S9). Over time, filaments aggregated into coherent, rotating assemblies that closely resembled the ACRs observed in vitro (Fig. 2*C* and Movie S7). These simulated rings also displayed stable, CW rotation and reproduced the experimentally observed transition from streaming filaments to ring formation. Thus, the combination of intrinsic curvature and interfilament interactions is sufficient to drive the self-organization of ACRs.

### Structural Characterization of ACRs.

The ACRs exhibited remarkable temporal and spatial stability. Once formed, they continued to rotate at fixed positions without detectable displacement (Fig. 2*B* and Movies S2, S3, and S7). Moreover, the ACRs maintained their ring-shaped architecture even after rotational motion ceased upon ATP depletion. Notably, ACRs were formed exclusively from highly purified actin and *Cc*XI MD (*SI Appendix,* Fig. S7), indicating that neither actin-bundling proteins nor other auxiliary factors were required for their formation. Taken together, these observations underscore the exceptional stability of the ACR as a self-organized structural unit composed solely of actin filaments.

**Fig. 3.**
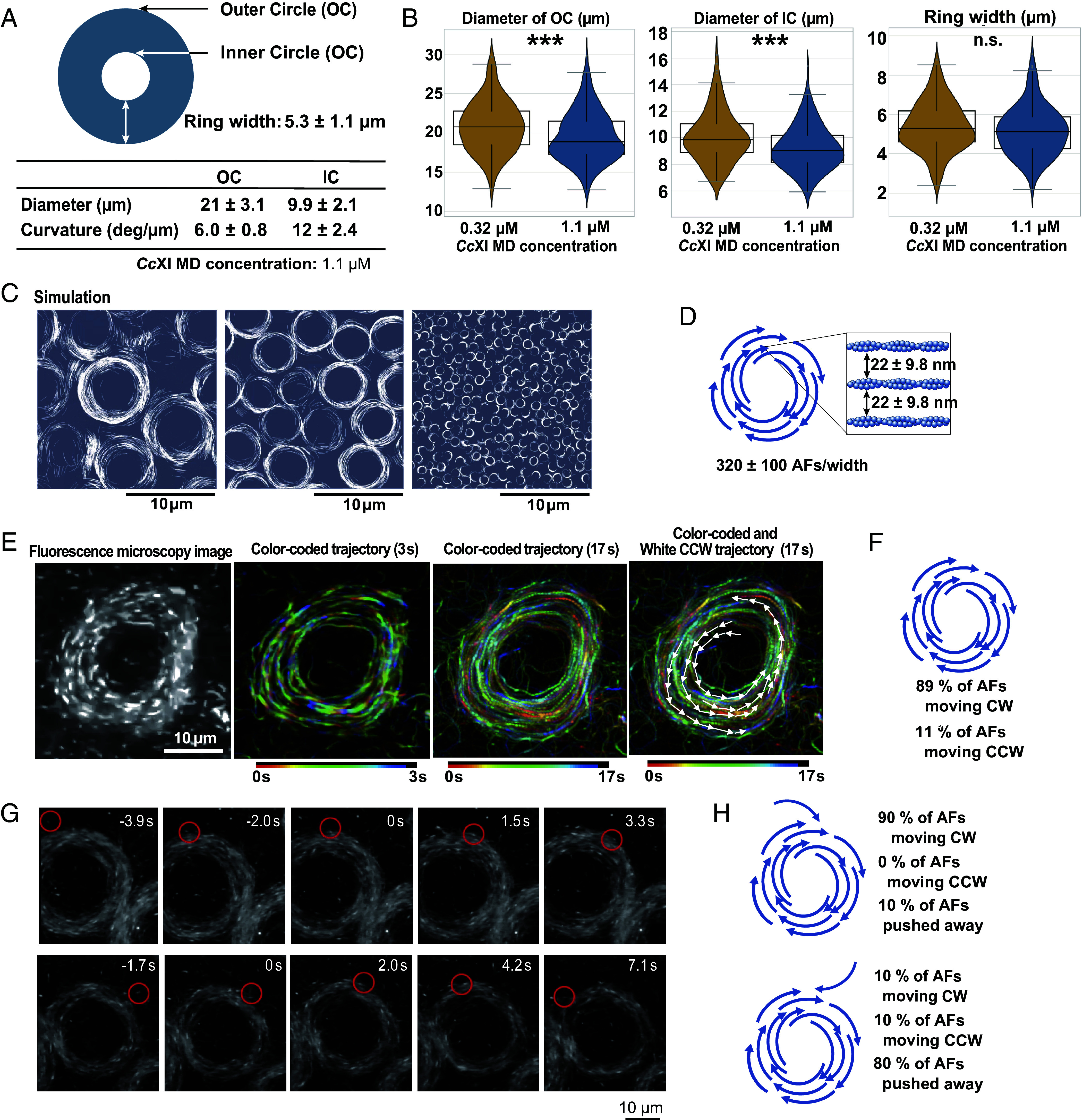
Analysis of ACR structures (*A*) Quantification of outer and inner diameters of ACR. Measurements were obtained from two independent protein preparations (*N* = 2), yielding a total of 280 ACRs (outer diameter: 21 ± 3.1 µm; inner diameter: 9.9 ± 2.1 µm). Values for each preparation were as follows: outer diameter = 20 ± 3.1 µm (*n* = 160) and 22 ± 2.4 µm (*n* = 120); inner diameter = 9.3 ± 1.7 µm (*n* = 120) and 11 ± 2.1 µm (*n* = 120). (*B*) Violin plots comparing geometric parameters of ACRs formed at 0.32 µM and 1.1 µM *Cc*XI MD. (*Left*) Outer diameter, (*Center*) Inner diameter, (*Right*) Ring width. Because ACR diameters show slight preparation-dependent variation, all measurements in this panel were obtained using the same protein preparation (*N* = 1) to ensure internally consistent comparison. Each violin shows 160 measurements per condition; medians are indicated by white dots and interquartile ranges by thick black bars. Statistical analysis was performed using the Mann–Whitney *U* test (*SI Appendix, Supplementary Materials and Methods*). Outer and inner diameters were significantly smaller at 1.1 µM (*U* = 15,327.5, *P* = 0.00027; *U* = 16,078.0, *P* = 4.6 × 10^−5^, respectively), whereas ring width showed no significant difference (*U* = 20,564.0, *P* = 0.099). Orange: 0.32 µM; Blue: 1.1 µM. (*C*) Simulations show that increased filament curvature (via decreased persistence length) produces smaller ACRs (Movie S10). (*D*) Sparse labeling allowed estimation of ~320 filaments per ring width, with average spacing of ~22 nm (*SI Appendix*, Fig. S8). (*E*) Fluorescence image (*Left*) and time color-coded trajectories of filaments within an ACR. The second and third panels show 3-s and 17-s trajectories, respectively, highlighting persistent circular motion. Most filaments rotated CW, whereas ~10% moved CCW; these CCW filaments are indicated by white arrows in the rightmost panel, which overlays the 17-s trajectory. This process is shown in Movie S11. (*F*) Directional analysis of 140 filaments from seven ACRs: 89% CW vs. 11% CCW (*G*) Representative time-lapse fluorescence microscopy images showing the collision of actin filaments with ACR. (*Upper*) A filament approaching the ACR from the same direction as its rotation is incorporated and adopts CW rotation (Movie S12). (*Lower*) A filament approaching from the opposite direction is repelled from the ACR (Movie S13). (*H*) Quantification of collision outcomes (*n* = 30 per approach direction). All experiments shown in this figure were conducted under the *Bausch condition* (moderately high–actin density in vitro motility assay).

ACRs displayed remarkable uniformity, with outer and inner diameters of 21 ± 3.1 µm and 9.9 ± 2.1 µm, respectively (Fig. 3*A*). The inner curvature (~12 deg/µm) closely matched the trajectory curvature of individual filaments (Fig. 1*C*), suggesting a shared mechanistic basis, whereas the outer curvature was markedly reduced, likely limiting expansion and ensuring size uniformity. Because the curvature of single actin filaments depends on surface myosin density (Fig. 1*D*), we next examined whether altering myosin density would also influence collective ACR organization. Increasing filament curvature by raising *Cc*XI MD concentration (0.32 → 1.1 µM) yielded smaller ACRs (Fig. 3*B*). This relationship was further evaluated by in silico simulations, in which the persistence length—a parameter that determines single-filament curvature—was modulated to alter curvature.

(*SI Appendix, Supplementary Materials and Methods, Simulation on Motility Assay of Multiple Actin Filaments*). The results demonstrated a clear trend: Filaments with greater curvature formed ACRs with smaller diameters (Fig. 3*C* and Movie S10), consistent with the experimental observations (Fig. 3*B*).

To determine the number of actin filaments per ring width within the ACR, we adjusted the ratio of Cy3-labeled to nonfluorescent actin (*SI Appendix*, Fig. S8) to enable clear visualization of each filament. We estimated the number of actin filaments per ring width within the ACR to be 320 ± 100 filaments corresponding to an interfilament spacing of approximately 22 ± 9.8 nm (Fig. 3*D*). This spacing—approximately three times the diameter of a single actin filament—suggests that filaments within the ACR are relatively separated rather than densely packed.

To analyze the dynamics of individual filaments within ACRs, we visualized sparsely labeled actin filaments prepared under the *Bausch condition*, using a very small amount (2.4 nM) of phalloidin–labeled fluorescent actin (*SI Appendix, Supplementary Materials and Methods, In Vitro Motility Assay under the Bausch Condition*). Time-lapse tracking revealed that each filament maintained a stable circular trajectory without deviating into adjacent paths, indicating that actin filaments rotate persistently within fixed lanes, thereby forming a coherent and stable rotational array. Most filaments moved CW, but some moved CCW (Fig. 3*E* and Movie S11). Directional analysis of 140 filaments from seven ACRs showed that the majority (89%) rotated CW, whereas a minority (11%) rotated CCW (Fig. 3*F*).

To examine how actin filaments are incorporated into ACRs, we analyzed collision events in which filaments approached either in the same direction as the ACR’s rotation or from the opposite direction. When filaments approached in the same direction, they were smoothly incorporated into the ring and adopted CCW rotation (Fig. 3 *G*, *Upper* and Movie S12). Among 30 filaments analyzed, 90% were incorporated with CCW rotation, while the remaining 10% were excluded (Fig. 3 *H*, *Upper*). By contrast, when filaments approached from the opposite direction, they were typically repelled upon contact and not incorporated (Fig. 3 *G*, *Lower* and Movie S13). Among 30 filaments analyzed under these conditions, 80% were repelled, 10% were incorporated and rotated in the CW direction, and the remaining 10% were incorporated but rotated in the CCW direction (Fig. 3 *H*, *Lower*). These latter CCW filaments likely correspond to the minority population observed within the ACR (Fig. 3*E*, four white arrows in the *Rightmost* panel).

### Modulation of ACR Formation by Actin-Bundling Factors.

In living cells, actin-binding factors frequently regulate filament organization by promoting actin bundling. To examine how bundling influences ACR dynamics, we tested two representative factors: villin, which directly bundles actin filaments, and methylcellulose (MC), which induces depletion-driven actin bundling while increasing solution viscosity.

In this study, we used *Arabidopsis thaliana* villin 1 (AtVLN1) as a representative villin. The lily villin P-135-ABP has been shown to bundle actin filaments with uniform polarities (30). In contrast, whether AtVLN1 bundles actin filaments in a polarity-aligned manner has not been directly measured. However, AtVLN1 localizes to actin filament bundles in plant cells, where filament polarity is known to be uniformly aligned, leading to the prediction that AtVLN1 likewise promotes polarity-aligned bundling (31). Previous reports demonstrated that actin filaments bundled by polarity-aligned bundling proteins move at velocities comparable to those of single filaments, whereas filaments bundled by polarity-random bundling factors exhibit little or no motility in the in vitro motility assays (32, 33). Consistent with this distinction, actin filaments bundled by AtVLN1 moved at velocities similar to those of single actin filaments (Movie S14), supporting the interpretation that AtVLN1 bundles actin filaments with uniform polarity.

**Fig. 4.**
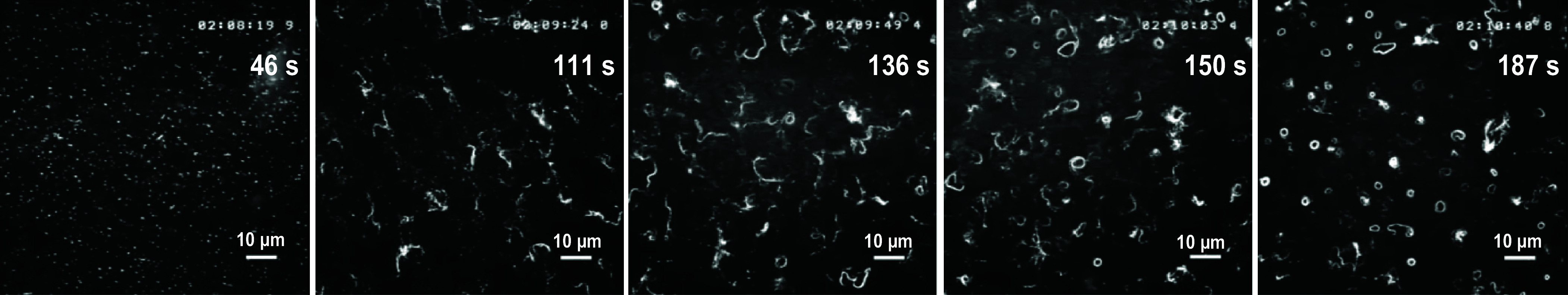
Time-lapse images of small ACRs formed in the presence of villin. Under the Bausch condition at an initial actin concentration of 2.4 µM, collective actin dynamics driven by *Cc*XI MD were examined after ATP perfusion supplemented with 1.6 µM *Arabidopsis thaliana* villin 1 (VLN1). Representative time-lapse snapshots shown here are extracted from Movie S14, illustrating the rapid emergence of numerous small ACRs (~5 µm in outer diameter) that rotate CW. The presence of villin markedly accelerated ACR formation, reducing the onset time from ~10 min to only a few minutes. Villin (VLN1) is known to bundle actin filaments with aligned polarities, thereby promoting a uniformly oriented filament population that facilitates coherent ring rotation. Consequently, villin-induced ACRs were substantially smaller than control ACRs (~20 µm) yet retained robust CW rotation.

In the presence of AtVLN1, ACR formation occurred within only a few minutes (Fig. 4 and Movie S14), markedly faster than under the same condition in the absence of AtVLN1, where ACRs typically appeared after ~10 min (Fig. 2*C* and Movie S7). This acceleration can be attributed to the ability of VLN1 to selectively bundle filaments with aligned polarities, thereby generating a uniformly oriented filament population required for coherent ACR rotation. The resulting ACRs were substantially smaller (~5 µm outer diameter, compared with ~20 µm without villin), yet maintained robust CW rotation. These results indicate that villin-mediated acceleration of ACR formation and reduction in ring size are likely due to its polarity-aligning bundling activity, without compromising the rotational stability or self-organizing capacity of ACRs (Fig. 4 and Movie S14).

By contrast, MC, which induces depletion-driven actin-bundling, destabilized rings (*SI Appendix*, Figs. S9 and S10 and Movies S15 and S16). When villin and MC were combined, villin counteracted the destabilizing effect of MC, resulting in the eventual emergence of small, stable ACRs (*SI Appendix*, Fig. S11 and Movie S17). These results suggest that protein-mediated bundling with uniform polarity can promote stable ACRs, whereas depletion-driven bundling at high filament densities destabilizes them. Detailed experimental conditions, results with interpretations, and quantitative analyses are provided in the *SI Appendix, *Supplementary Text**. A systematic summary of the experimental conditions and the resulting collective actin behaviors observed in the presence of villin and/or MC is provided in *SI Appendix*, Table S2.

## Discussion

This study reveals a mode of actin self-organization driven by chiral myosin activity. We found that *Cc*XI, a fast plant myosin XI, drives actin filaments in the CW direction. At high actin density, this activity induces the autonomous formation of ACRs through collective motion. Remarkably, ACRs are exceptionally stable, maintaining continuous rotation at their formation sites.

### Mechanism of Myosin-Driven Chiral Actin Motion.

The trajectories of the leading and trailing ends of *Cc*XI MD–driven chiral curved actin filaments were identical (Fig. 1*E*), indicating that the chiral curvature originates specifically at the filament tip. This geometric profile suggests that macroscopic curvature results not from a singular, large-scale rotational event, but from the cumulative effect of incremental directional deflections occurring as the filament tip repeatedly encounters surface-bound myosin heads. Based on this cumulative deflection model, an increase in myosin surface density is predicted to enhance filament curvature by increasing the frequency of these tip-myosin encounters (*SI Appendix,* Fig. S1). Our experimental finding that actin filament curvature scaled positively with myosin density (Fig. 1*D*) provides strong evidence for this frequency-dependent, tip-localized mechanism.

A fundamental question arises: Why is lateral bending confined exclusively to the filament tip? While myosin heads interact with actin along its entire length and exert oblique forces globally, the internal regions of the filament are mechanically constrained by rigor-bound myosins situated both anterior and posterior to any active power-stroking motor. As illustrated in *SI Appendix*, Fig. S2, these rigor-state myosins function as mechanical “pegs” that effectively anchor the internal segments and restrict their lateral displacement. In contrast, the leading tip is free from forward mechanical constraints, allowing it to respond to the off-axis component of the myosin power stroke with lateral bending. The accumulated angular changes at the tip, followed by the trailing body, ultimately yield the persistent chiral trajectories observed in our assays (*SI Appendix*, Fig. S2).

What mechanism underlies the chiral curved motion of the filament’s leading tip? The widely accepted lever-arm model posits that myosin moves actin filaments via a swing of its lever arm during the power stroke (34). If the lever arm of *Cc*XI swings at an oblique angle relative to the filament axis, this could account for the observed curvature. To explore this possibility, we compared the rigor-state structures of acto-*Cc*XI MD, resolved by cryoelectron microscopy (35), with that of acto-SkII subfragment 1, also determined by cryoelectron microscopy (36). Structural analysis revealed that the lever arm positions of *Cc*XI and SkII in the rigor state were nearly identical, both aligning parallel to the actin filament (*SI Appendix*, Fig. S12). Thus, the postpower stroke conformation cannot account for the chiral curved motion.

Recent cryo-EM analysis of mouse myosin IC provides valuable insight into how chiral force generation might arise. That study demonstrated that the lever arm of myosin IC is tilted in the ADP-bound (prepower stroke) state but becomes nearly parallel in the rigor state, suggesting that an oblique power stroke originates from a prepower stroke conformation (37). By analogy, *Cc*XI may also adopt an oblique lever arm orientation in the prepower stroke state, generating the directional bending force responsible for chiral curved motion. One of the main structural determinants of the altered lever-arm orientation of myosin IC in the ADP-bound state was attributed to differences in its actin-binding interface (37). On the basis of this finding, we compared the actin-binding residues of *Cc*XI and SkII, which revealed substantial differences not only in the amino acids of the myosin binding loops but also in the corresponding actin residues (*SI Appendix*, Table S3). These variations may, as in the case of myosin I, underlie conformational changes in the prepower stroke state that contribute to the emergence of chiral curved motion.

### Collective Actin Dynamics and ACR Formation.

Our results indicate that ACR formation is a density-selective mode of collective actin dynamics rather than a generic outcome of high actin concentration. Under the Bausch condition, which removes filaments from solution and restricts dynamics to the surface, robust ACR formation occurred within a moderately high actin density regime (~15 filaments/µm^2^). In this regime, ACRs emerged with high frequency and within approximately 10 min after ATP perfusion (Fig. 2*C* and Movie S7). By contrast, skeletal muscle myosin II systems at comparable filament densities have been reported to predominantly transition into polar cluster states, rather than forming closed assemblies (12). Thus, although filament density acts as a shared control parameter across myosin systems, the collective state selected at this density differs between myosin classes, with *Cc*XI favoring closed chiral assemblies rather than extended polar flows.

Across all conditions, the earliest collective state observed after ATP addition was nematic streaming, whose orientation consistently aligned with the direction of solution inflow (Figs. 2*C*, *SI Appendix*, Figs. S4 and S5, and Movies S4–S7). This correspondence strongly suggests that nematic alignment is imposed by perfusion-induced hydrodynamic flow rather than arising spontaneously from actomyosin interactions. As filament density increased from the moderately high to the high actin density regime, this flow-imposed alignment became increasingly stable, prolonging nematic streaming and biasing collective dynamics. Under the Bausch condition within the high actin density regime (~40 filaments/µm^2^), nematic and polar streaming phases were prolonged, and ACR formation became delayed and less frequent (*SI Appendix*, Fig. S4 and Movie S4). Upon further increasing filament density into the ultra–high actin density regime (~75 filaments/µm^2^, near-saturated surface coverage), nematic streaming persisted for extended periods and was frequently followed by polar streaming, with ACRs appearing only sporadically and after substantial delays (*SI Appendix*, Fig. S5 and Movie S5).

A similar dominance of robust, flow-aligned streaming was observed under the Molloy condition, which likewise corresponds to an ultra–high actin density regime. However, in this Molloy condition, in addition to the surface being near-saturated (~70 filaments/µm^2^), a large excess of filaments remains in the bulk solution. This situation likely stabilizes long-lasting nematic streaming, thereby preventing transitions to polar waves or ACR formation (*SI Appendix*, Fig. S6 and Movie S6). Together, these observations suggest that strong uniaxial alignment—whether reinforced by high or ultra–high actin density at the surface or by filaments persisting in solution—biases collective dynamics toward open streaming states and kinetically suppresses global reorganization into closed rings. A systematic overview of the actin density regimes, experimental conditions, and the resulting collective states discussed here is provided in *SI Appendix*, Table S1.

In this view, the reduced frequency and delayed appearance of ACRs observed in the high and ultra–high actin density regimes reflect not only steric crowding but also the stabilizing influence of perfusion-imposed nematic alignment and a preferential nematic-to-polar transition pathway. Reducing directional flow during actin introduction may therefore weaken initial nematic alignment and expand the parameter space permissive for ACR formation, even under conditions corresponding to bulk actin concentrations approaching physiological levels.

### Mechanism of ACR Formation.

To understand the mechanism behind ACR formation, it is informative to consider prior studies on actin self-organization driven by SkII. Under similar actin densities, SkII promotes the emergence of polar collective actin states. It has been proposed that straight-moving filaments gradually align in parallel by interacting with neighboring filaments, whereby multiple filament interactions collectively guide their leading ends into alignment (13, 14). By analogy, one could hypothesize that if the same principle were applied to chiral curved filaments, ACR formation would follow naturally (Fig. 2*D*). Indeed, the real-time imaging of ACR formation (Fig. 2*C* and Movie S7) closely resembles the sequence depicted in this schematic.

We simulated multifilament interactions using a simplified model in which each filament’s leading edge adjusted its orientation according to nearby filaments. Strikingly, simulations with straight-moving filaments produced only nematic alignment (Movie S8), consistent with earlier studies that polarity bias is required for polar streaming (13, 14). By contrast, when intrinsic curvature was introduced, filaments spontaneously organized into coherent polar arrays that closed into rotating rings (Movie S9). These simulations resembled the experimental transition from nematic streaming to ACR formation (Movie S7). Importantly, both simulations and experiments revealed a consistent relationship: Higher filament curvature yielded smaller ACR diameters (Fig. 3 *B* and *C* and Movie S10). This agreement provides strong support that multifilament interactions, combined with tip-driven curvature, constitute the mechanistic basis of ACR formation. The synergy between in vitro assays and modeling thus highlights a general framework for how chiral active matter can self-organize into robust rotational structures. Note, however, that ACR formation is not expected for all myosins with chiral activity; rather, a sufficiently strong chiral activity—specifically, a threshold level of tip-induced curvature in a consistent direction—appears to be required.

Interestingly, similar chiral rotating ring structures have been observed in the bacterial cytoskeletal system. FtsZ, a tubulin homolog, forms curved filaments that undergo chiral treadmilling when attached to lipid membranes via FtsA. At high densities, these filaments self-organize into rotating chiral rings in vitro (22, 23), and simulations support the autonomous formation (20, 23). Notably, this system also involves intrinsically chiral curved filaments that, through filament–filament interactions, assemble into rotating ring structures—a process strikingly similar to the ACR formation revealed in our study. Taken together, these parallels raise the possibility that chiral ring formation through collective motion may represent a fundamental and evolutionarily conserved mechanism of cytoskeletal organization. Comparable chiral rings have also been reported for malaria parasites; although they display a clear chirality bias, their origin lies not in molecular or filament-level chirality, as in FtsZ or ACRs, but in cell morphology and gliding motility (38).

Beyond these microbial and parasite systems, ring-like collective dynamics have also been reported in cytoskeletal assemblies based on actin and microtubules. In a previous study using SkII, the addition of fascin, an actin-bundling protein that aligns filament polarities, induced rotating actin rings without the involvement of chiral motors (39, 40). This demonstrates that polarity-aligned bundling alone can generate circular trajectories. A key distinction, however, is the rotational bias: Fascin-induced rings exhibited both CW and CCW rotations with no preference, whereas *Cc*XI-driven rings in our study consistently rotated in the CW direction. In dynein-driven microtubule assays, collective dynamics gave rise to large vortices in which filaments circulated both CW and CCW without chirality bias (41). In kinesin-driven microtubule assays, MC promoted the formation of CCW-rotating rings whose chirality arises from the intrinsic left-handed supertwist of microtubules (42). Unlike these systems, where either no chirality or microtubule-intrinsic twist combined with external crowding agents defined the outcome, ACRs emerge solely from actin filaments propelled by a chiral myosin, highlighting a uniquely minimal system in which motor chirality alone establishes a persistent CW bias. These comparisons highlight that, unlike previously reported systems, the CW bias observed here originates from the intrinsic chirality of *Cc*XI rather than from bundling effects or filament supertwist.

### Potential Roles of ACRs in Cell Dynamics.

Although the physiological roles of ACRs remain largely unexplored, several possible functions can be inferred from previous in vivo observations and from the collective dynamics characterized here.

ACR-like structures, often referred to as acquosomes, have been reported in diverse plant cells, including *Arabidopsis*, tobacco BY2 cells, zinnia, and *Characean algae*. These structures have been suggested to participate in the formation of uniformly polarized actin filament bundles that underlie processes such as cytoplasmic streaming and pollen tube elongation (43–51). In extracts from *Characean algae*, these structures rotated on glass surfaces in the same direction as the ACRs characterized here, and electron microscopy showed that they consist of uniformly polarized actin bundles (47), suggesting that in vivo acquosomes may share structural and dynamic properties with in vitro ACRs. Collectively, these observations suggest that ACRs may provide an experimentally tractable in vitro analog of acquosomes in plant cells.

Many animal cells also exhibit intrinsic L–R chirality driven by chiral actin organization (52–54), which can propagate to tissue-scale asymmetry and organ morphogenesis (7, 55–58). Notably, *Drosophila* myosin ID (Dm ID), which shares functional similarity with *Cc*XI, induces chiral curved motion of actin filaments in vitro (4) and is essential for establishing cellular and organ-level asymmetry (4–7). In our recent preprint, we showed that *Dm* ID forms ACRs in vitro and that overexpression of *Dm* ID in *Drosophila* hemocytes—cells that normally express little endogenous *Dm* ID—drives directional actin flows matching the ACR rotation (25). These findings suggest that *Dm* ID can directly link molecular-scale chirality to cell-scale chirality through the induction of ACR-like structures. Because *Dm* ID exhibits slow motility, ACR formation in vitro requires extended time, which precluded detailed observation of the formation process and filament dynamics within the rings (25). Nevertheless, given its shared property of driving chiral filament motion, it is likely that *Dm* ID forms ACRs through a mechanism analogous to that revealed here for *Cc*XI.

## Materials and Methods

See also *SI Appendix, *Supplementary Materials and Methods**.

### Protein Expression and Purification.

*Cc*XI MD and *Cb*XI-3 MD were expressed in insect cells (High Five™, Life Technologies) and purified using nickel-affinity and FLAG-affinity resins as previously described (28, 29). Further details are described in the *SI Appendix*, Materials and Methods**. Skeletal muscle myosin II was purified from rabbits as described previously (59, 60). Skeletal muscle actin was purified from chicken breast muscle using the method of Spudich and Watt (61). Cy3-labeled skeletal muscle actin was prepared as described previously (60, 62).

## Supplementary Material

Appendix 01 (PDF)

Movie S1. **Chiral curved motion of actin filaments driven by *Cc*XI MD** This movie shows the chiral curved motion of actin filaments driven by *Cc*XI MD in the standard *in vitro* motility assay. Actin filaments moved clockwise (CW) when viewed from the objective lens side (myosin side). Experimental conditions are described in *SI Appendix, Supplementary Materials and Methods*, “Standard *in vitro* motility assay”. The movie is shown in real time. Scale bar, 10 µm.

Movie S2. **Clockwise rotation of ACRs formed by *Cc*XI MD under the Bausch condition at an initial actin concentration of 2.4 µM (moderately high actin density)**. This movie shows the formation of actin chiral rings (ACRs) through collective filament motion under the Bausch condition at the initial actin concentration of 2.4 µM. ACRs emerged approximately 10 min after ATP addition and subsequently exhibited stable clockwise (CW) rotation at their formation sites. Experimental conditions are described in *SI Appendix, Supplementary Materials and Methods*, “*in vitro* motility assay under the Bausch condition” and summarized in Table S1. The movie is shown at 5× speed. Scale bar, 10 µm. (Related to Fig. 2B.)

Movie S3. **Clockwise rotation of actin ACRs formed by *Cc*XI MD under the Bausch condition at the initial actin concentration of 2.4 μM (moderately high actin density) in 150 mM KCl**. This movie shows CW-rotating ACRs formed under the Bausch condition at an initial actin concentration of 2.4 μM in 150 mM KCl (physiological ionic strength). ACRs formed and rotated stably, indicating that ACR formation is not restricted to the standard 25 mM KCl buffer. Experimental conditions are described in *SI Appendix, Supplementary Materials and Methods*, “*in vitro* motility assay under the Bausch condition”. The movie is shown at 10× speed. Scale bar, 10 μm.

Movie S4. **Time-resolved transitions from nematic and polar streaming to ACR formation by *Cc*XI MD following ATP perfusion under the Bausch condition at the initial actin concentration of 7.1 μM (high actin density)**. This movie illustrates the time-resolved collective dynamics of actin filaments after ATP perfusion in the *in vitro* motility assay under the Bausch condition using an initial actin concentration of 7.1 μM unlabeled actin. Because the collective behaviors evolved over time and prolonged imaging at a single location caused significant photobleaching, each characteristic state was recorded at a different field of view within the same chamber, capturing representative dynamics at defined time points. Immediately after ATP addition, actin filaments exhibited nematic streaming, characterized by bidirectional alignment and flow (120–145 s). The orientation of this nematic streaming coincided with the direction of solution inflow into the chamber. As the system evolved, orientational symmetry gradually broke, and polar streaming emerged (480–550 s), characterized by unidirectional filament motion. At later time points, ACRs formed and stabilized (1,350–1,450 s). Compared with the moderately high– and high–actin density conditions (the Bausch condition at an initial actin concentration of 2.4 μM (Movie S7), corresponding to a filament density of 15 ± 4.1 filaments/μm^2^, ACR formation required a longer time to occur, and the frequency of ACR appearance was notably lower under this higher actin density condition (41 ± 3.1 filaments/μm^2^). The nematic, polar, and ACR states shown here represent distinct collective regimes selected to visualize the temporal evolution of actin self-organization following ATP perfusion. Experimental conditions are described in *SI Appendix, Supplementary Materials and Methods*, “*in vitro* motility assay under the Bausch condition,” and summarized in Table S1. The movie is shown at 10× speed. Scale bar, 10 μm. (Related to Fig. S4.)

Movie S5. **Time-resolved transitions from nematic and polar streaming to ACR formation by *Cc*XI MD following ATP perfusion under the Bausch condition at the initial actin concentration of 24 μM (ultra–high actin density)**. This movie illustrates the time-resolved collective dynamics of actin filaments after ATP perfusion in the *in vitro* motility assay under the Bausch condition using an initial concentration of 24 μM unlabeled actin. Because prolonged imaging at a single location caused photobleaching, each characteristic state was recorded from a different field of view within the same chamber. Immediately after ATP addition, actin filaments displayed nematic streaming (40–72 s), with the flow direction aligned with the direction of solution inflow. As time progressed, polar streaming became the dominant and persistent collective mode, appearing repeatedly across different locations (1,010–1,080 s and 2,190–2,250 s). ACRs first appeared at later time points (1,990–2,090 s). Notably, even after ACRs emerged, polar streaming persisted at other locations within the same chamber (e.g., 2,239 s), indicating that polar motion remained a prevalent collective state. At later times (2,360–2,400 s), ACRs were again observed at distinct positions, demonstrating that under this higher actin density condition, at which actin filaments were saturated at the surface, ACRs and persistent polar streaming coexisted spatially rather than converging into a single global pattern. Compared with the Bausch condition at an initial actin concentration of 2.4 μM (Movie S7) corresponding to a filament density of 15 ± 4.1 filaments/μm^2^, this higher actin density condition (75 ± 10 filaments/μm^2^) exhibited a markedly prolonged predominance of polar streaming, resulting in delayed and infrequent ACR formation. The movie is shown at 10× speed. Scale bar, 10 μm. (Related to Fig. S5.)

Movie S6. **Time-resolved persistent nematic streaming of actin filaments driven by *Cc*XI MD under the Molloy condition at the initial actin concentration of 24 μM**. This movie shows time-lapse observations of actin filaments after perfusion of a solution containing 24 μM unlabeled actin filaments together with ATP into a chamber coated with *Cc*XI MD (Molloy condition). To assess the reproducibility and spatial robustness of the collective dynamics, imaging was performed at four distinct fields of view within the same chamber. Throughout the ~1,600-s observation period, actin filaments aligned along their longitudinal axes and moved bidirectionally, a hallmark of nematic streaming, without forming higher-order structures such as actin chiral rings (ACR). Although the initial filament alignment reflected the direction of solution inflow, nematic streaming persisted even as local filament orientations varied across different regions of the chamber, confirming that this behavior reproducibly occurred at multiple locations on the same coverslip. Experimental conditions are described in the *SI Appendix, Supplementary Materials and Methods*, “*in vitro* motility assay under the Molloy condition,” and summarized in Table S1. The movie is shown at 20× speed. Scale bar, 10 μm. (Related to Fig. S6.)

Movie S7. **Continuous real-time process of ACR formation by *Cc*XI MD following ATP perfusion under the Bausch condition at the initial actin concentration of 2.4 μM (moderately high actin density)**. This movie captures the dynamic process of ACR formation following ATP addition: initial stream-like filament flow, coalescence into nascent rings, and maturation into stable ACRs, which rotated CW. Experimental conditions are described in *SI Appendix, Supplementary Materials and Methods*, “*in vitro* motility assay under the Bausch condition” and summarized in Table S1. The movie is shown at 20× speed, corresponding to 40–650 s after ATP addition. Scale bar, 10 μm. (Related to Fig. 2C.)

Movie S8. **Simulation of collective motion of straight actin filaments**. This movie illustrates a simulation of collective motion of straight actin filaments forming nematic streams. The filaments align along their longitudinal axes via nematic interactions, resulting in bidirectional streaming. The color represents the sine of the angle between each filament’s movement direction and the x-axis (sin θ). Blue indicates motion with negative sin θ, and yellow indicates motion with positive sin θ. This behavior reflects the fundamental characteristics of nematic alignment in straight filament systems (see *SI Appendix, Supplementary Materials and Methods*, “Simulation on motility assay of multiple actin filaments”). This movie is played at a rate of 15 s of simulation time per second of real time. Scale bar = 10 μm.

Movie S9. **Simulation of collective motion of chiral curved actin filaments (persistence length = 10 μm)**, This movie illustrates a simulation of collective motion of chiral curved actin filaments, leading to the formation of ACRs (persistence length = 10 μm). This simulation corresponds to the control condition used for comparison in Movie S9. This control simulation replicates experimental observations and demonstrates that the collective motion of filaments with chiral curved motion results in the formation of ACRs (see *SI Appendix, Supplementary Materials and Methods*, “Simulation on motility assay of multiple actin filaments”). The color represents the sine of the angle between each filament’s movement direction and the x-axis (sin θ); blue indicates negative sin θ and yellow indicates positive sin θ. This movie is played at a rate of 15 s of simulation time per second of real time. Scale bar = 10 μm. (Related to Fig. 2E.)

Movie S10. **Simulations of collective motion of chiral curved filaments with different individual curvatures**. This movie presents side-by-side simulations comparing the collective motion of chiral curved actin filaments with different degrees of single-filament curvature: left, low curvature; center, intermediate curvature; right, high curvature (see *SI Appendix, Supplementary Materials and Methods*, “Simulation on motility assay of multiple actin filaments”). In these simulations, the curvature of individual filaments is controlled by the persistence length, a parameter that determines single-filament curvature. Specifically, filament curvature was increased by reducing the persistence length (Lp): left, Lp = 10 μm (same simulation as Movie S8); center, Lp = 5 μm; right, Lp = 1 μm. In all panels, filaments undergo chiral curved motion and spontaneously self-organize into rotating actin chiral rings (ACRs). As the curvature of individual filaments increases, the diameter of the resulting ACRs becomes progressively smaller.The color represents sin θ (blue, negative; yellow, positive). Playback rates are as follows: left and center panels, 15 s of simulation time per 1 s of real time; right panel, 1.5 s of simulation time per 1 s of real time. Scale bar, 10 μm. (Related to Fig. 3C.)

Movie S11. **Fluorescence imaging of individual actin filaments within a fully formed ACR**. This movie shows trajectories of individual filaments within a fully formed ACR. Most filaments rotated CW, while ~10% rotated CCW. Filaments maintained stable, lane-preserving trajectories throughout the observation period. Compared with Movies S2–S6, a smaller amount of fluorescent actin was added during the observation phase, which reduced overall fluorescence but allowed clearer visualization of individual filament trajectories. Experimental conditions are described in *SI Appendix, Supplementary Materials and Methods*, “*in vitro* motility assay under the Bausch condition” and summarized in Table S1. The movie is shown at 2× speed. Scale bar, 10 μm. (Related to Fig. 3E.)

Movie S12. **Collision of an actin filament with an ACR from the same direction as the ring’s rotation**. This movie shows a filament colliding with an ACR from the same direction as the ring’s CW rotation. The filament was incorporated and adopted CW rotation. Compared with Movies S2–S6, a smaller amount of fluorescent actin was added during the observation phase, which reduced overall fluorescence but allowed clearer visualization of individual filament trajectories. Experimental conditions are described in *SI Appendix, Supplementary Materials and Methods*, “*in vitro* motility assay under the Bausch condition” and summarized in Table S1. The movie is shown in real time. Scale bar, 10 μm. (Related to Fig. 3G, upper panel.)

Movie S13. **Collision of an actin filament with an ACR from the opposite direction to the ring’s rotation**. This movie shows a filament colliding with an ACR from the opposite direction to its CW rotation. The filament was repelled without incorporation. Compared with Movies S2–S6, a smaller amount of fluorescent actin was added during the observation phase, which reduced overall fluorescence but allowed clearer visualization of individual filament trajectories. Experimental conditions are described in *SI Appendix, Supplementary Materials and Methods*, “*in vitro* motility assay under the Bausch condition” and summarized in Table S1. The movie is shown in real time. Scale bar, 10 μm. (Related to Fig. 3G, lower panel.)

Movie S14. **Effect of villin on ACR formation**. This movie shows the accelerated formation of small ACRs (~5 μm in outer diameter) driven by *Cc*XI MD in the presence of 1.6 μM villin under the Bausch condition at an initial actin concentration of 2.4 μM. Within a few minutes after ATP perfusion, numerous small ACRs rapidly formed, whereas under the same condition without villin, ACR formation typically required ~10 min. Thus, villin accelerates ACR formation by several fold. Once formed, the small ACRs continued to rotate clockwise (CW) at fixed positions. A larger field of view than in other movies was recorded to capture more ACRs within a single frame. Experimental conditions are described in *SI Appendix, Supplementary Materials and Methods* (“*in vitro* motility assay under the Bausch condition”) and summarized in Table S2. The movie is shown at 20× speed, corresponding to 40–290 s after ATP addition. Scale bar, 25 μm. (Related to Fig. 4.)

Movie S15. **Stability of ACRs formed in the presence of increasing methylcellulose concentrations under the Bausch condition**. This movie compiles representative time-lapse recordings of actin chiral rings (ACRs) driven by *Cc*XI MD in the presence of 0.25%, 0.5%, or 1.0% methylcellulose (MC) under the Bausch condition at an initial actin concentration of 2.4 μM (moderately high actin density). At 0.25% MC, ACRs exhibited stable clockwise (CW) rotation. At 0.5% MC, some ACRs disassembled during observation, indicating reduced stability. At 1.0% MC, ACR formation was infrequent and instability was more pronounced, with frequent disassembly events. Representative time-lapse snapshots shown in Fig. S9 are extracted from this movie. Experimental conditions are described in *SI Appendix, Supplementary Materials and Methods*, “*in vitro* motility assay under the Bausch condition,” and summarized in Table S2. The movie is shown at 10× speed. Scale bar, 10 μm. (Related to Fig. S9.)

Movie S16. **Effect of methylcellulose on ACR formation under the Hatori condition**. This movie shows the effect of 0.5% methylcellulose under the Hatori condition. Actin filaments (2.4 μM) were infused into a chamber coated with *Cc*XI MD, following the same loading protocol as the Bausch condition used for the moderately high–actin concentration. However, under the Hatori condition, the unbound-actin washing step was omitted to slightly increase the effective actin filament concentration near the surface, thereby enhancing methylcellulose-mediated depletion effects that promote actin filament bundling (10). Subsequently, an ATP solution containing 0.5% methylcellulose was added. To characterize the temporal evolution of actin organization, representative time-lapse images capturing the most characteristic structures and dynamics at each time point were recorded at four distinct fields of view within the same chamber (22–222 s, 490–630 s, 644–818 s, and 840–1115 s after ATP and methylcellulose addition). Immediately after the addition of ATP and methylcellulose, actin filaments exhibited nematic flow. Approximately 10 min after ATP and methylcellulose addition, large vortices (~100 μm) appeared. These vortices rotated clockwise (CW) but typically disassembled within a few minutes. Experimental conditions are described in the *SI Appendix, Supplementary Materials and Methods*, “in vitro motility assay under the Hatori condition for methylcellulose assays,” and summarized in Table S2. The movie is shown at 20× speed. Scale bar, 10 μm. (Related to Fig. S10.)

Movie S17. **Combined effects of villin and methylcellulose on ACR formation under the Hatori condition**. This movie shows the combined effects of 1.6 μM villin and 0.5% methylcellulose under the Hatori condition. Actin filaments (2.4 μM) were infused into a chamber coated with *Cc*XI MD, following the same loading protocol as the Bausch condition used for the moderately high– actin concentration. However, under the Hatori condition, the unbound-actin washing step was omitted to slightly increase the effective actin filament concentration near the surface, thereby enhancing methylcellulose-mediated depletion effects that promote actin filament bundling (10). Subsequently, an ATP solution containing 1.6 μM villin and 0.5% methylcellulose was added. To characterize the temporal evolution of actin organization, representative time-lapse images capturing the most characteristic structures and dynamics at each time point were recorded at four distinct fields of view within the same chamber (60–95 s, 565–603 s, 1,605–1,645 s, and 2,014–2,074 s after ATP addition).Immediately after ATP, villin, and methylcellulose addition, actin filaments exhibited nematic flow, which gradually transitioned into unstable large rings that typically disassembled by ~1,000 s. Subsequently, stable small ACRs (~5 μm in diameter), comparable to those formed in the presence of villin alone (Fig. S8 and Movie S14), began to appear. Experimental conditions are described in the *SI Appendix, Supplementary Materials and Methods*, “*in vitro* motility assay under the Hatori condition for methylcellulose assays,” and summarized in Table S2. The movie is shown at 5× speed. Scale bar, 10 μm. (Related to Fig. S11.)

## Data Availability

Study data are included in the article and/or supporting information.
