# Supplementary material for: Elucidating chiral myosin–induced actin dynamics: From single-filament behavior to collective structures
Source: Proc Natl Acad Sci U S A. 2026 Jan 28;123(5):e2508686123. doi: 10.1073/pnas.2508686123 (PMC12867714; doi:10.1073/pnas.2508686123)
Supplement: Supplementary file 1 — Appendix 01 (PDF) [file pnas.2508686123.sapp.pdf]

PNAS

Supporting Information for
Elucidating Chiral Myosin-Induced Actin Dynamics: From Single Filament Behavior to Collective Structures

Takeshi Haraguchi, Kohei Yoshimura, Yasuhiro Inoue, Takuma Imi, Koyo Hasegawa, Taisei Nagai, Hideki Furusawa, Toshifumi Mori, Kenji Matsuno and Kohji Ito

Corresponding author: Kohji Ito, Takeshi Haraguchi
Email: k-ito@faculty.chiba-u.jp

This PDF file includes:

Supplementary Materials and Methods (pages 2–7)
Supplementary Text (pages 8–9)
Figures S1 to S12 (pages 10–21)

Tables S1 to S3 (pages 22–24)
Legends for Movies S1 to S17 (pages 25–29)
SI References (pages 30–31)

Other supporting materials for this manuscript include the following:

Movies S1 to S17

Supplementary Materials and Methods

Protein engineering

*Cc*XI MD （*Chara corallina* Myosin XI）

A baculovirus transfer vector for *Cc*XI MD (pFastBac *Cc*XI MD) was generated as follows: The cDNA encoding the motor domain of *Chara corallina* myosin XI (amino acid residues 1–746; UniProt ID: Q9LW97) was synthesized with codons optimized for Trichoplusia ni (Eurofins Genomics). This sequence corresponds to a motor domain with a lever arm length of approximately 3.5 nm. The construct was digested with BamHI and AgeI and ligated into a similarly digested pFastBac *Cb*XI-1 MD vector(1). The resulting vector, pFastBac *Cc*XI MD, encodes the following structure:

·N-terminal tag: MDYKDDDDKRS (includes a FLAG tag: DYKDDDDK)

·Motor domain: residues 1–746 of Chara corallina myosin XI

·C-terminal tag:

GGGEQKLISEEDLHHHHHHHHSRMDEKTTGWRGGHVVEGLAGELEQLRARLEHHPQGQREPSR (comprising a flexible linker GGG, Myc epitope EQKLISEEDL, $(His)_8$ tag, and SBP tag)

This construct is identical in motor domain sequence to that used in previous studies (2-4), with the exception of N- and C-terminal tag sequences.

*Cb*XI-3 MD （*Chara braunii* Myosin XI-3）

A baculovirus transfer vector for *Cb*XI-3 MD (pFastBac *Cb*XI-3 MD) was generated as previously described (1). The construct encodes the motor domain of *Chara braunii* myosin XI-3 (amino acid residues 1–747; UniProt ID: A0A388L5N7), which also corresponds to a lever arm length of approximately 3.5 nm. The complete construct has the following configuration:

·N-terminal tag: MDYKDDDDKRS (includes a FLAG tag: DYKDDDDK)

·Motor domain: residues 1–747 of *Chara braunii* myosin XI-3

·C-terminal tag:

GGGEQKLISEEDLHHHHHHHHSRMDEKTTGWRGGHVVEGLAGELEQLRARLEHHPQGQREPSR (comprising a flexible linker GGG, Myc epitope EQKLISEEDL, $(His)_8$ tag, and SBP tag)

This construct is identical to the one used in our previous publication (1).

Baculovirus transfer vectors of pFastBac *Cc*XI MD and *Cb*XI-3 MD were expressed in insect cells (High Five^{TM}, Life Technologies) and the expressed proteins were purified using nickel–affinity and FLAG-affinity resins as previously described (1, 4).

AtVillin 1

The full-length cDNA of *Arabidopsis thaliana* Villin 1 (gene: AT2G29890.1, UniProt ID: O81643) was provided by the RIKEN BRC through the National BioResource Project of MEXT/AMED, Japan (RIKEN Arabidopsis full-length cDNA clone, resource number: pda11214). The cDNA was subcloned into the pRSET expression vector (Invitrogen, Carlsbad, CA, USA), which contains an N-terminal His-tag. *Escherichia coli* strain BL21 (DE3) was transformed with the resulting expression plasmid. Transformed cells were cultured in LB broth supplemented with 50 µg/ml ampicillin at 20°C until the optical density at 600 nm (OD600) reached 0.6. Protein expression was induced by adding 0.4 mM IPTG, followed by incubation at 16°C for 16 hours. The *E. coli* cells expressing AtVillin 1 were harvested by centrifugation at 1,400 × *g* for 12 minutes at 4°C. The cell pellet was resuspended and lysed in lysis buffer containing 300 mM KCl, 0.1 mM EGTA, 10 mM β-mercaptoethanol (β-ME), 0.5% Triton X-100, 2 mg/ml lysozyme, 10 mM HEPES-KOH (pH 8.0), and a protease inhibitor cocktail (50 µg/ml leupeptin, 5 µg/ml pepstatin A, 0.25 mM PMSF, and 5 µg/ml chymostatin). The cells were flash-frozen in liquid nitrogen, thawed, and subjected to sonication to ensure complete lysis. The lysate was centrifuged at 38,000 × *g* for 30 minutes at 4°C. The resulting supernatant was incubated with nickel-nitrilotriacetic acid (Ni-NTA) agarose (Qiagen) on a rotating wheel for 1.5 hours at 4°C. The resin suspension was loaded onto a plastic column and washed with 200 ml of wash buffer containing 300 mM KCl, 10% glycerol,

15 mM imidazole, 10 mM β-ME, 10 mM HEPES-KOH (pH 8.0), and the same protease inhibitor cocktail. A second wash was performed using 15 ml of buffer containing 10% glycerol, 15 mM imidazole, 10 mM β-ME, and the protease inhibitor cocktail. AtVillin 1 was eluted with buffer containing 10% glycerol, 200 mM imidazole, 10 mM β-ME, and the same protease inhibitors.

Standard *in vitro* motility assay

The standard *in vitro* motility assay was used for Fig.1–E and Movie S1 and performed as previously described (1, 4). Briefly, 1.3 μM of anti-c-Myc antibody was perfused into the motility chamber. After washing with assay buffer, 1.1 μM of myosin molecules with a c-Myc epitope tag were perfused into the chamber to immobilize the myosin molecules on the coverslip surface using an anti-c-Myc antibody. Next, the assay chamber was infused with either assay buffer containing 8 nM rhodamine-phalloidin-labeled actin or the same concentration of phalloidin-labeled Cy3-actin. The composition of the assay buffer for observation was as follows: 25 mM HEPES-KOH (pH 7.4), 25 mM KCl, 4 mM $MgCl_2$, 3 mM ATP, 10 mM DTT, and an oxygen scavenging system (0.75 μM glucose oxidase, 12.8 mM glucose, and 0.08 μM catalase). Observations of motility were conducted at 25°C.

***in vitro* motility assay under the Bausch condition**

The Bausch condition was used for Figs. 2B, 2C, 3A, 3B, 3D–3H, 4, and S3–S5, S8, and S9; and Movies S2–S5, S7, and S11–S15. This assay was performed essentially as described by Bausch et al. (5) and consisted of four main steps. Unlabeled actin filaments were introduced at initial concentrations of 2.4 μM, 7.1 μM, or 24 μM together with a small amount of fluorescently labeled actin under ATP-free conditions, allowing filaments to bind to surface-immobilized myosin. This loading procedure yields a markedly higher effective density of surface-bound filaments than that typically achieved in standard *in vitro* motility assays using 10 nM fluorescently labeled actin.

1. Myosin immobilization

   Myosin molecules were immobilized on the coverslip of the chamber using an anti-c-Myc antibody as described in the standard *in vitro* motility assay (1, 3, 4, 6, 7).

2. Actin loading

   For experiments performed at an initial actin concentration of 2.4 μM, ATP-free assay buffer containing 2.4 μM unlabeled actin together with a defined amount of fluorescently labeled actin was introduced into the chamber (Figs. 2B, 2C, 3A, 3B, 3D–3H, 4, and S3, S8, S9, and Movies S2, S3, S7, and S11–S15).

   The type and concentration of fluorescently labeled actin were varied depending on the experiment, as follows. Rhodamine–phalloidin–labeled actin was added at 8 nM for Figs. 2B, 2C, 3A, 3B, and S9, and Movies S2, S3, S7, and S14–S15. For experiments requiring reduced background fluorescence (Figs. 3E–3H, Movies S11–S13), 2.4 nM rhodamine–phalloidin–labeled actin was used. Alternatively, phalloidin–Cy3–labeled actin was added at 2.4 nM for Figs. 3D, S3, and S8.

   For actin introduced at an initial concentration of 7.1 μM, ATP-free assay buffer containing 19 nM phalloidin–Cy3–labeled actin and 7.1 μM unlabeled actin was introduced into the chamber (Fig. S4; Movie S4).

   For actin introduced at an initial concentration of 24 μM, ATP-free assay buffer containing 19 nM phalloidin–Cy3–labeled actin and 24 μM unlabeled actin was introduced into the chamber (Fig. S5; Movie S5).

   For Cy3 labeling, actin was conjugated with Cy3-succinimide. To avoid motility artifacts, the fraction of Cy3-labeled actin incorporated into filaments was kept below 10%, as previously reported (8). Unlabeled actin filaments were shortened by either gelsolin treatment or sonication prior to use (see below, *Filament length control*).

3. Removal of actin filaments not bound to myosin

   After a 10-min incubation of fluorescently labeled actin (rhodamine–phalloidin or Cy3) and 2.4 μM, 7.1 μM, or 24 μM unlabeled actin in the chamber, actin filaments that were not bound to surface-immobilized myosin were removed by washing with 10 chamber volumes of ATP-free assay buffer.

4. Initiation of motility

ATP-containing assay buffer (3 mM ATP) for observation was then introduced to initiate filament motility and imaging. When the aim was to observe ring structure formation rather than filament movement (Fig. 2B, 2C, S4, and S5; Movies S2, S3–S5, and S7), a lower concentration of fluorescent actin (~4 nM rhodamine-phalloidin-labeled actin) was added to the ATP-containing assay buffer during the observation phase. In contrast, a very small amount (~1 nM rhodamine-phalloidin-labeled actin) was added to the ATP-containing assay buffer to avoid background signal interference (Fig. 3E–3G; Movies S11–S13). When the number of actin filaments was estimated using Cy3-actin, no additional fluorescent actin was added (Figs. 3D, S3 and S8).

Composition of assay buffer for observation

The composition of the assay buffer for observation (ATP-containing assay buffer, 3 mM ATP) was the same as that used for the standard *in vitro* motility assay—except that, a small amount of rhodamine–phalloidin–labeled actin was included (see above) and for Movie S3, which was conducted using 150 mM KCl instead of 25 mM KCl. Motility observations were conducted at 25 °C.

* For Fig. 4 and Movie S14, the assay buffer for observation additionally contained 1.6 μM villin.
* For Fig. S9 and Movie S15, the assay buffer for observation contained 0.25%, 0.5%,or 1.0% methylcellulose.

in vitro motility assay under the Molloy condition

The Molloy condition was used for Fig. S6 and Movie S6. This assay was performed essentially as described by Molloy et al. (9) with the following two steps:

1. Myosin immobilization
   Myosin molecules were immobilized on the coverslip using an anti-c-Myc antibody, as in the standard *in vitro* motility assay.
2. Initiation of motility
   ATP-containing assay buffer (3 mM ATP) for observation was introduced to initiate motility and imaging. Unlabeled actin filaments were shortened by either gelsolin treatment or sonication prior to use (see below, *Filament length control*).

A summary of the collective actin movement modes observed under the actin density condition, together with the corresponding figures and movies, is provided in *SI Appendix,* Table S1.

in vitro motility assay under the Hatori condition for methylcellulose assays

The Hatori condition for methylcellulose assays was used for Figs. S10 and S11 and Movies S16 and S17. This assay was identical to that under the Bausch condition, except that the unbound-actin washing step was omitted to slightly increase the effective actin filament concentration near the surface, thereby enhancing methylcellulose-mediated depletion effects that promote actin filament bundling (10), and consisted of the following three steps:

1. Myosin immobilization
   Myosin molecules were immobilized on the coverslip of the chamber using an anti-c-Myc antibody as described in the standard *in vitro* motility assay (1, 3, 4, 6, 7).
2. Actin loading
   For actin introduced at an initial concentration of 2.4 μM, ATP-free assay buffer containing 8 nM rhodamine–phalloidin–labeled actin together with 2.4 μM unlabeled actin was introduced into the chamber.
3. Initiation of motility
   ATP-containing assay buffer (3 mM ATP) for observation was then introduced to initiate filament motility and imaging. For Fig. S10 and Movie S16, 0.5% methylcellulose was included in the ATP-containing assay buffer. For Fig. S11 and Movie S17, both 0.5% methylcellulose and 1.6 μM villin were included.

Filament length control

In the high–actin density *in vitro* motility assays for collective motion, unlabeled actin filaments were shortened either by gelsolin treatment or by sonication:

- Gelsolin treatment: Actin was incubated with gelsolin (Sigma-Aldrich, product number: G8032) at a molar ratio of 1000:1 (actin : gelsolin) in the presence of 1 mM $CaCl_2$, yielding filaments with an average length of approximately 5 µm. Movies S2, S4, S5, S6, and S15–S17; Figs. 2B, 2C, S4, S6, and S9 show experiments performed with gelsolin-treated actin filaments.
- Sonication: Actin samples (1000 µL) were sonicated using a UD-200 ultrasonic disruptor (Tomy Seiko Co., Ltd., Tokyo, Japan) equipped with a TP-030 microtip (2 mm diameter), set at intensity level 2. Sonication was performed on an ice-water bath in continuous pulse mode for 10 seconds, followed by a 2-minute cooling period. This cycle was repeated three times for a total sonication time of 30 seconds. The resulting actin filaments were fragmented to an average length of approximately 5 µm, as confirmed by fluorescence microscopy. Movies S3, S11–S14, S18, and S19; Figs. 3E, 3G, S8, S10, and S11 show experiments performed with sonicated actin filaments.

Notably, ACRs also formed even when actin filaments were not pre-treated with either gelsolin or sonication. In these cases, longer actin filaments were initially present but were gradually severed over time during the *in vitro* motility assay, eventually reaching shorter lengths. As a result, the formation of ACRs was slightly delayed.

Measuring the curvature of the trajectory of a single actin filament in the standard *in vitro* motility assay

An orientation difference-based method was used to calculate curvature from a set of points. Specifically, the curvature was calculated by dividing the orientation angle differences by the distance between two points using the orientation information of the point sequence (11). The X and Y coordinates of the leading end of a single actin filament were measured at 1 µm intervals over 21 points using Bohboh Soft (12). We then determined the curvature of the trajectory of a single actin filament from a set of 21 points spaced 1 µm apart by dividing the total angular variation by the total distance of movement across these 21 points. For each myosin type studied, curvature measurements were taken from approximately 30 actin filaments and then averaged to obtain the final values.

Simulation on motility assay of multiple actin filaments

To perform simulations on motility assay of multiple actin filaments, we construct a mathematical model to express the movement of the actin filament. In the model, each actin filament consists of n_s segments of which length is σ. Assuming that the leading tip of the i-th actin filament could be followed by the rest of the segments, the movement of the filament can be described in terms of the leading tip motion using the following equation of motion (13):

$$x_i(t + \Delta t) = x_i(t) + v_{\mathrm{a}}\Delta t \cos(\theta_i + \Delta\theta_i) \quad (1)$$

$$y_i(t + \Delta t) = y_i(t) + v_{\mathrm{a}}\Delta t \sin(\theta_i + \Delta\theta_i) \quad (2)$$

where x_i and y_i are the x and y components of the position vector of the leading tip of the i-th actin filament, respectively. In the model, the sliding velocity of each actin filament is expressed by the average sliding velocity, v_{a}, and the direction of the motion of the i-th actin filament is expressed by the angle θ_i measured counterclockwise from the x axis on x −y plane. The change of moving direction of the leading tip of the i-th actin filament, $\Delta\theta_i$, is calculated by the following equation:

$$\Delta\theta_i(t) = \frac{\delta^2 f_{\mathrm{myo}} v_{\mathrm{a}} \Delta t}{3 k_{\mathrm{B}} T L_p} + \dot{\Theta}_{\mathrm{nem}}(\theta_i)\Delta t. \quad (3)$$

Here, f_{myo} is a myosin-induced active force normal to the filament axis at the tip of the i-th actin filament, δ is the mean interspace of myosin heads on the substrate, k_{B} is Boltzmann constant, T is the temperature. $\dot{\Theta}_{\mathrm{nem}}(\theta_i)$ is a directional change rate of the tip per unit time through nematic interactions between actin filaments. To express $\dot{\Theta}_{\mathrm{nem}}(\theta_i)$ based on a filament alignment interaction, we refer to an energy function describing the intermolecular pairwise interaction of nematic liquid crystal (14). By differentiating this energy function with respect to the angle difference, $\dot{\Theta}_{\mathrm{nem}}(\theta_i)$ is derived as

$$\dot{\Theta}_{\mathrm{nem}}(\theta_i) = \frac{1}{\tau}\sum_j \cos(\theta_j - \theta_i)\sin(\theta_j - \theta_i) \quad (4)$$

where the relaxation time, τ, is introduced to express kinetics of the filament alignment in our model. The summation in the right-hand side of Eq. (4) is applied over the j-th segment of a filament if the j-th segment of the filament is located within a cut-off distance of $\sigma/2$ from the the tip of the i-th filament, and the angle difference $\left|\theta_j - \theta_i\right|$ exceeds the nematic threshold angle θ_c. Because we allow filaments to be overlapped in the simulations, we ignored the explicit contribution of the inverse of the distance between filaments such as a weight of interaction.
To examine the influence of filament curvature on ACR formation, we systematically varied the persistent length (L_p) of actin filaments in the simulations. The persistence length is a physical parameter that reflects the bending stiffness of a filament and determines the extent of its trajectory curvature. In the simulations, intrinsic chiral curvature was implemented as a constant torque at the filament tip, while the persistence length modulated how prominently this curvature manifested. Unless otherwise stated, the standard L_p was set to 10 μm; for increased curvature, it was reduced to 5 μm (Movie S8) or 1 μm (Movie S9).
The model parameters are listed below.

- Filament density: 12.5 filaments/μm^2
- Filament length: 1 μm
- Filament velocity: 2 μm/s
- Myosin spacing: 0.005 μm/molecule
- Mean velocity of sliding motion of the filament: 2 μm/s
- Persistence length of the filament: 10 μm
- Length of the coarse-grained filament segment: 5 x 10^{-2} μm
- The number of segments of the filament: 20

- Temperature: 310 K
- Normal force density acting on the filament tip: -2 x 10^{-2} μN/μm
- Relaxation time for aligning filaments: 1 x 10^{-2} s
- Nematic threshold angle: 0.451 rad

Periodic boundaries are adopted for *x* and *y* directions, where the unit box size is $20 \times 20\ \mu m^2$.

Preparation of Methylcellulose Solutions for Cytoplasmic Viscosity Simulation
Because quantitative information for plant cells is scarce, we surveyed measurements in animal cells. Intracellular viscosity varies substantially with subcellular location, probe size, and measurement scale. In animal cells, bulk cytoplasmic viscosity is typically ~10–100 cP at 25 °C (10–100× that of water), whereas aqueous microdomains can be close to water-like (~1–1.5 cP), and cytoskeleton-rich regions can reach ~1,000–10,000 cP due to macromolecular crowding (15-17). Direct viscosity data for plant cells are limited, but reported values are of similar magnitude, with local increases in cytoskeleton- and organelle-rich regions.

To mimic these intracellular viscous environments in vitro, we prepared methylcellulose (MC) solutions at 0.25%, 0.5%, and 1.0% (w/v) using MC powder (Sigma-Aldrich, M0512; CAS No. 9004-67-5) with a nominal viscosity of ~4,000 cP at 2% in water at 25 °C. This specific grade has been employed in rheological studies, and the measured values were ~20.6 cP for 0.49% MC and ~79 cP for 0.83% MC (18). We interpolated other concentrations under the assumption of an approximately logarithmic relationship between viscosity (η) and MC concentration (C), expressed as log η ≈ a·C + b (19). Using the direct measurements (18) and the approximation (19), the viscosity values were estimated as follows

- 0.25% MC: ~8 cP
- 0.5% MC: ~21 cP
- 1.0% MC: ~150 cP

These viscosities span the physiological range of bulk cytoplasm (~10–100 cP at 25 °C), thereby enabling simulation of different intracellular viscosity conditions in motility assays.

Quantification of ACR geometry
Outer diameter, inner diameter, and ring width of ACRs were quantified from fluorescence microscopy images under two myosin concentrations (0.32 μM and 1.1 μM *Cc*XI MD). For each

condition, 160 measurements were obtained. Statistical comparisons were carried out using the Mann–Whitney U test to evaluate differences between the two concentrations. Results are presented in Fig. 3B as violin plots, showing medians, interquartile ranges, and overall distributions.

Statistical analysis

For single filament curvature (Fig. 1D), trajectories of 30 actin filaments were analyzed for each myosin surface density condition. Because multiple groups were compared, we applied one-way ANOVA followed by Tukey's HSD post hoc test to assess statistical significance. Significant differences between groups are indicated in Fig. 1D.

For geometric parameters of ACRs (Fig. 3B), outer diameter, inner diameter, and ring width were measured at 0.32 μM and 1.1 μM CcXI MD ($n = 160$ each). Statistical comparisons were performed using the Mann–Whitney U test. The results revealed significant differences in both outer diameter ($U = 15,327.5$, $p = 0.00027$) and inner diameter ($U = 16,078.0$, $p = 4.6 \times 10^{-5}$), while ring width showed no significant difference ($U = 20,564.0$, $p = 0.099$).
All statistical analyses were performed using Python's scipy.stats module. Figures 1D and 3B illustrate the results as box plots and violin plots, respectively, showing medians, interquartile ranges, and data distributions.

Supplementary Text

Effects of methylcellulose (MC) on ACR formation under the *Bausch condition*

MC was used at 0.25%, 0.5%, and 1.0% (w/v). The corresponding viscosities of the same MC product (Sigma-Aldrich, M0512; CAS No. 9004-67-5), estimated from published rheological data, span the physiological range of bulk cytoplasm (~10–100 cP at 25 °C) (*SI Appendix, Supplementary Materials and Methods*, "Preparation of Methylcellulose Solutions for Cytoplasmic Viscosity Simulation"). Importantly, at concentrations above ~0.2% (w/v), MC functions not only as a viscosity enhancer but also as a promoter of actin filament bundling through depletion effects (20, 21). Actin bundles begin to appear at ~0.25% MC, and at 0.5% a more extensive bundle network is typically observed. Similar depletion forces are also expected to arise *in vivo* owing to the highly crowded molecular environment of the cytoplasm, although their precise contribution to actin bundling *in vivo* remains unresolved because of the concurrent actions of actin-binding proteins, ionic conditions, and other cytoskeletal regulators.

Under the *Bausch condition* with MC, ACRs were formed and rotated CW as in the absence of MC. However, ACRs formed in the presence of MC tended to appear sharper, with narrower ring widths than those without MC. Moreover, we frequently observed the disappearance of ACRs during imaging, a phenomenon never detected without MC. Both the sharpening of ring structures and the instability of ACRs became more pronounced at higher MC concentrations. These MC-induced changes in ACR morphology and stability were more evident as the MC concentration increased (*SI Appendix,* Fig. S9; Movie S15). This result demonstrates that ACRs can form under viscosities comparable to those in the cytoplasm. However, they also reveal that increasing MC concentration renders ACRs progressively more unstable.

Effects of methylcellulose on ACR formation under the Hatori condition

The instability of ACRs induced by MC may be attributable to two factors: increased viscosity and enhanced filament bundling due to depletion effects. It is known that depletion-induced bundling by MC becomes more effective at higher actin concentrations, even at the same MC concentration (22). To distinguish whether instability arises primarily from viscosity or from depletion effects, we maintained MC at 0.5% and increased actin density by switching from the *Bausch condition* (with washing) to Hatori condition. This assay was identical to that under the Bausch condition, except that the unbound-actin washing step was omitted to slightly increase the effective actin filament concentration near the surface, thereby enhancing methylcellulose-mediated depletion effects that promote actin filament bundling (10), (*SI Appendix, Supplementary Materials and Methods, "in vitro* motility assay under the Hatori condition for methylcellulose assays," Table S2).

Under these conditions, very thick actin bundles were observed to move actively, a phenomenon essentially identical to that reported when similar conditions were applied using skeletal muscle myosin II (10). Typical ACRs were not observed. Here, the indicated time corresponds to the approximate onset of vortex formation rather than ATP perfusion. Occasionally, large CW-rotating vortices with diameters up to ~100 μm appeared transiently but disintegrated within 1–a few minutes, and new ones repeatedly formed at different locations, only to vanish again, reflecting highly transient and unstable dynamics at this stage (*SI Appendix,* Fig. S10; Movie S16). Thus, although ACRs could still form under the Bausch condition at the same MC concentration (*SI Appendix,* Fig. S9; Movie S15), they failed to appear under the *High–actin condition*, indicating that the primary destabilizing influence of MC is not viscosity itself, but rather depletion-induced filament bundling enhanced at higher actin densities.

Combined effects of villin and MC on ACR formation under the Hatori condition

When VLN1 and MC were combined under the Hatori condition, collective dynamics were markedly altered. Nematic streaming dominated initially, followed by unstable ~30 μm rings. These large rings disappeared within 1–a few minutes, and new ones repeatedly formed at different locations, only to vanish again. Strikingly, ~1000 s after ATP addition, stable small ACRs (~5 μm), comparable to those formed with villin alone, began to appear and persisted with continuous CW rotation. These small ACRs were comparable in size and stability to those formed with villin alone (Fig. 4; Movie

S14). By ~2000 s, large unstable rings had largely disappeared and ceased to reappear, leaving predominantly villin-like ACRs (*SI Appendix,* Fig. S11; Movie S17). These results indicate that villin can partially counteract the destabilizing influence of MC by fostering polarity-sorted bundles that support the formation of small, stable rings.

Summary

Together, these results demonstrate that bundling factors exert contrasting effects on ACR dynamics. Villin accelerates ACR formation and favors small, stable rings, while MC destabilizes ACRs through depletion-induced bundling at high filament densities. When combined, villin and MC produce a dynamic interplay that initially yields unstable large rings but ultimately results in stable villin-like ACRs. A systematic summary of the experimental conditions and outcomes in the presence of villin and/or methylcellulose is provided in the *SI Appendix*, Table S2.

Supplementary Figures

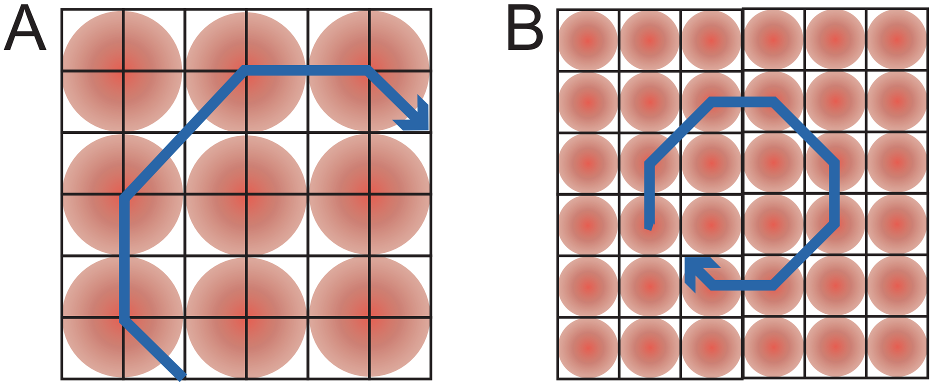

Fig. S1. Model illustrating the dependence of actin filament curvature on myosin density. In both panels, myosin motors are positioned at the center of circular areas. (A) Low-density condition: Nine myosin motors are distributed within the unit area, resulting in a relatively low myosin density. (B) High–density condition: Thirty-six myosin motors occupy the same unit area, increasing the myosin density by a factor of four compared to panel A. In both conditions, actin filaments interact with myosin at their leading tips, where oblique power strokes induce angular displacements of 45° in the clockwise (CW) direction. Although the angular displacement per stroke is identical in both panels, the overall curvature of actin motion increases under high–density conditions, as evident from the comparison between panels A and B. This enhanced curvature arises from the increased frequency of tip deflections due to more frequent myosin encounters at higher surface density.

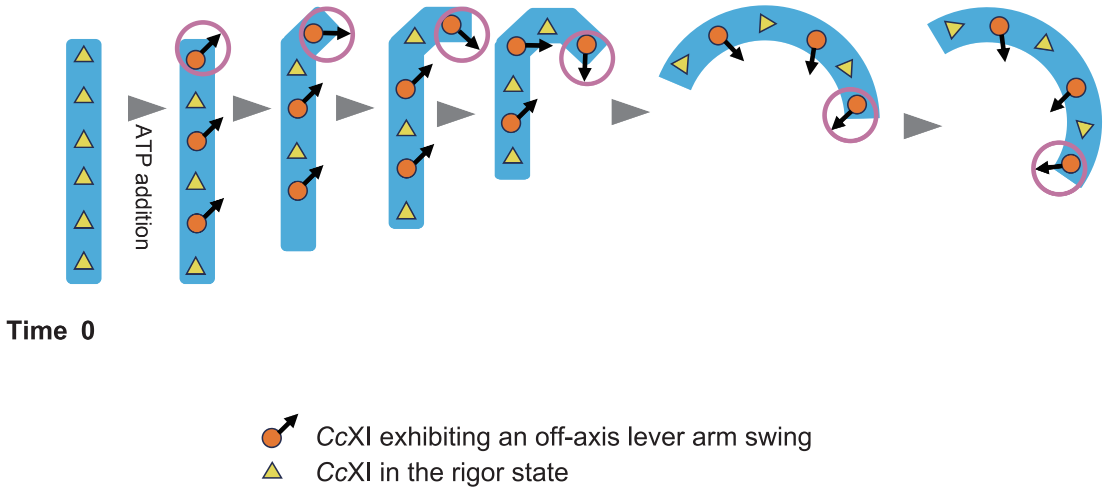

Fig. S2. Working hypothesis explaining why only the tip of actin filaments undergoes bending. The tip of an actin filament is free from mechanical constraints and can bend in response to the oblique component of the myosin power stroke. In contrast, internal regions of the filament are mechanically anchored by rigor-bound myosins located both ahead of and behind the power-stroking myosin. These rigor myosins act as pegs that restrict lateral displacement, effectively preventing bending in the off-axis direction. Although oblique power strokes occur along the entire filament, only the tip can respond with lateral bending due to the absence of such anchoring. The schematic at "Time 0" represents the point at which actin motility was initiated by ATP addition. At this point, only the tip deflects rightward in response to the oblique power stroke (indicated by red circles), while internal regions remain constrained. The filament tip is repeatedly deflected in the same direction. The rear segment itself does not bend but simply follows the tip. However, because the tip undergoes repeated deflections, the angular change accumulates with each cycle, ultimately leading to the formation of arc-shaped, chiral trajectories.

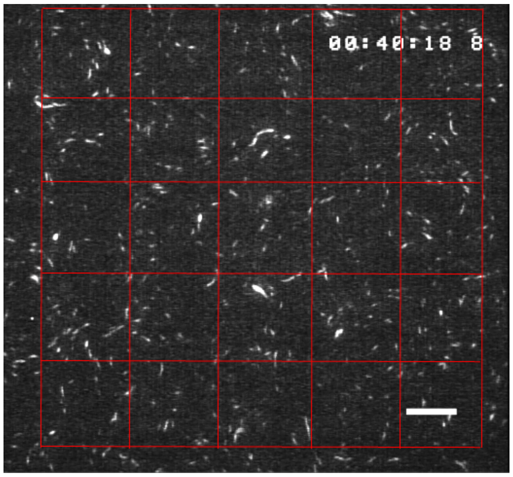

Fig. S3. Estimation of actin filament density in the *in vitro* motility assay under the Bausch and the Molloy condition. To estimate the surface density of actin filaments during collective motion in the *in vitro* motility assay under the *Bausch* and *Molloy condition,* we conducted experiments using a reduced fraction of fluorescently labeled actin. Unlabeled actin filaments were introduced at initial concentrations of 2.4 μM, 7.1 μM, or 24 μM together with a small amount of Cy3-labeled actin under ATP-free conditions (*SI Appendix, Supplementary Materials and Methods*, "in vitro motility assay under the *Bausch condition*)"). Consistent with the conditions used for ACR observation, actin filaments were pre-treated with gelsolin to control filament length.

For the 2.4 μM unlabeled actin condition, 8 nM Cy3-labeled actin was used, yielding a labeling ratio of approximately 1:300. For the 7.1 μM unlabeled actin condition, 19 nM Cy3-labeled actin was used, yielding a labeling ratio of approximately 1:375. For the 24 μM unlabeled actin condition, the same concentration of Cy3-labeled actin (19 nM) was used, corresponding to a labeling ratio of approximately 1:1,250. After a 10-min incubation in the absence of ATP, unbound actin filaments were removed by washing with ten chamber volumes of ATP-free buffer. Motility was then initiated by the addition of ATP, and imaging was started immediately.

The number of Cy3-labeled actin filaments per unit area was counted immediately after the onset of observation—prior to ACR formation—and multiplied by the corresponding labeling ratio to estimate the total number of filaments. Quantification was performed across ten fields of view (90 μm × 90 μm), each subdivided into 25 squares (18 μm × 18 μm).

In the representative field shown for the 2.4 μM condition in this figure. 337 Cy3-labeled filaments were detected immediately after ATP addition, corresponding to an estimated total of 337 × 300 = 101,100 filaments. This yields a filament density of 101,100 filaments/8,100 μm² = 12.5 filaments/μm². The average filament densities under the *Bausch condition* were 15 ± 4.1 filaments/μm² (n = 10), 41 ± 3.1 filaments/μm² (n = 6), and 75 ± 10 filaments/μm² (n = 6) for initial actin concentrations of 2.4 μM, 7.1 μM, and 24 μM, respectively. Under the *Molloy condition*, the average filament density was 71 ± 8.5 filaments/μm² (n = 6) for an initial actin concentration of 24 μM. Scale bar, 10 μm.

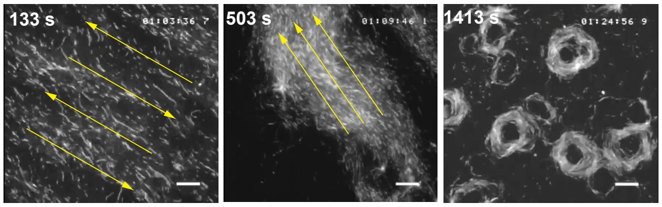

Fig. S4. Representative snapshots of sequential transitions from nematic and polar streaming to ACR formation after ATP perfusion under the Bausch condition at an initial actin concentration of 7.1 μM (high actin density).

This figure presents representative still images extracted from Movie S4 acquired using an initial actin concentration of 7.1 μM unlabeled actin under the Bausch condition. Because prolonged imaging at a single location caused significant photobleaching, each time point was recorded at a different field of view within the same chamber, capturing the characteristic collective states as they emerged over time. At 133 s, actin filaments exhibited nematic streaming, displaying bidirectional alignment and flow oriented along the direction of solution inflow. At 503 s, the system transitioned into polar streaming, characterized by unidirectional filament motion. At 1,413 s, actin chiral rings (ACRs) appeared and stabilized. Under this higher actin density condition (41 ± 3.1 filaments/μm²), ACR formation occurred later and with a lower frequency than under the moderately high–actin density condition (15 ± 4.1 filaments/μm²), corresponding to an initial actin concentration of 2.4 μM (Movie S7 and Fig. 2C). These snapshots correspond to the time windows highlighted in Movie S4 and illustrate the temporal progression from nematic to polar streaming and ultimately to ACR formation following ATP perfusion. Yellow arrows schematically indicate the direction of collective filament motion at each stage. Experimental conditions are described in the *SI Appendix, Supplementary Materials and Methods, “in vitro* motility assay under the Bausch condition,” and summarized in Table S1.Scale bar, 10 μm.

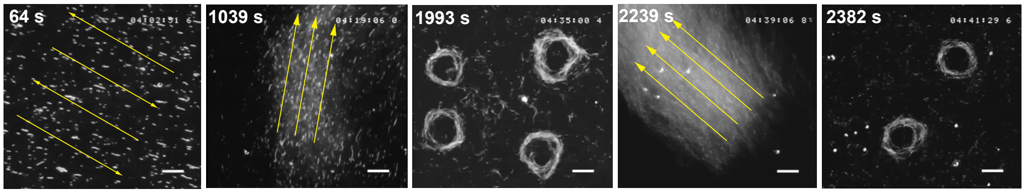

Fig. S5. Representative snapshots of altered sequential transitions from nematic and polar streaming to ACR formation after ATP perfusion under the Bausch condition at an initial actin concentration of 24 μM (ultra–high actin density).
This figure shows representative still images extracted from Movie S5 acquired using an initial actin concentration of 24 μM unlabeled actin under the Bausch condition. Because prolonged imaging at a single location caused photobleaching, each time point was recorded at a different field of view within the same chamber to capture the characteristic collective states. At 64 s, actin filaments exhibited nematic streaming, with the flow direction aligned with solution inflow. By 1,039 s, the system transitioned to polar streaming, which became the dominant and persistent collective mode. At 1,993 s, ACRs were observed, marking the onset of ring-shaped organization under this condition. At 2,239 s, polar streaming was again observed at a separate location, indicating that polar motion remained prevalent even after ACR formation. At 2,382 s, ACRs were present at distinct positions within the same chamber, demonstrating that under this high–actin density condition (75 ± 10 filaments/μm²), ACRs and persistent polar streaming coexisted spatially rather than forming a single unified pattern. ACR formation occurred substantially later and at a lower frequency than under the moderately high–actin density conditions, (15 ± 4.1 filaments/μm²), corresponding to an initial actin concentration of 2.4 μM (Movie S7 and Fig. 2C). Yellow arrows schematically indicate the direction of collective filament motion at each stage. Experimental conditions are described in the *SI Appendix, Supplementary Materials and Methods, "in vitro* motility assay under the Bausch condition," and summarized in Table S1.Scale bar, 10 μm.

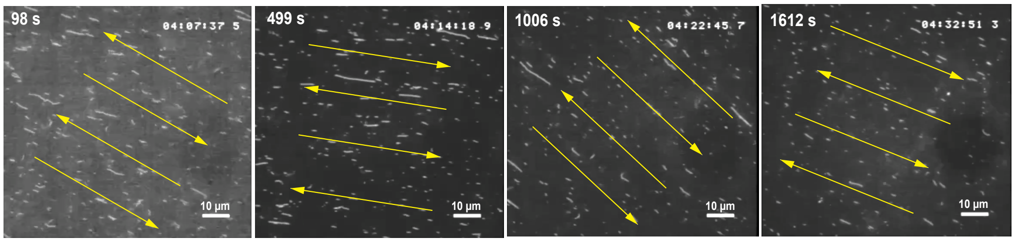

Fig. S6. Representative snapshots illustrating persistent nematic streaming of actin filaments after ATP perfusion under the Molloy condition at an initial actin concentration of 24 µM (ultra–high actin density).
This figure shows representative still images extracted from Movie S6 acquired using an initial actin concentration of 24 µM unlabeled actin under the Molloy condition. Immediately after perfusion of a solution containing 24 µM unlabeled actin filaments together with ATP into the chamber, actin filaments became aligned parallel to the direction of chamber perfusion, likely reflecting hydrodynamic effects during solution inflow. As observed previously for skeletal muscle myosin II under the Molloy condition, filament motion was bidirectional rather than polar, indicating nematic streaming (98 s). Representative still images extracted from Movie S6 at 98 s, 499 s, 1006 s, and 1612 s are shown; each snapshot corresponds directly to the time-lapse sequence presented in Movie S6. The indicated times correspond to the elapsed time after introduction of the actin/ATP solution. Each panel shows a different field of view on the same coverslip. Over an observation period of approximately 30 min, the preferred orientation of filaments varied across regions of the chamber, whereas the mode of collective motion consistently remained nematic, with no formation of higher-order structures such as actin chiral rings. Yellow arrows schematically indicate bidirectional (nematic) filament motion.
Experimental conditions are described in the *SI Appendix, Supplementary Materials and Methods, “in vitro* motility assay under the Molloy condition,” and summarized in Table S1. Scale bar, 10 µm.

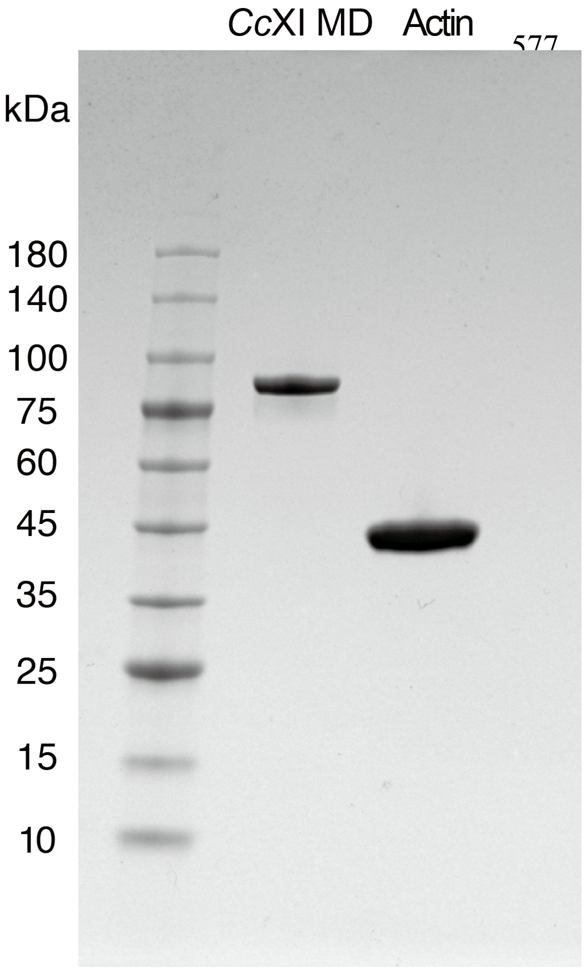

Fig. S7. SDS-PAGE analysis of purified *Cc*XI MD and actin.

Purified *Cc*XI MD and actin were analyzed by SDS-PAGE using a 4–20% polyacrylamide gradient gel and stained with Coomassie Brilliant Blue. Molecular mass markers (kDa) are indicated on the left. Both proteins displayed sharp and distinct bands, indicating near-homogeneous purity with no detectable contamination. These results confirm that the formation of actin chiral rings (ACRs) observed in this study was not attributable to the presence of other proteins, such as actin-bundling proteins.

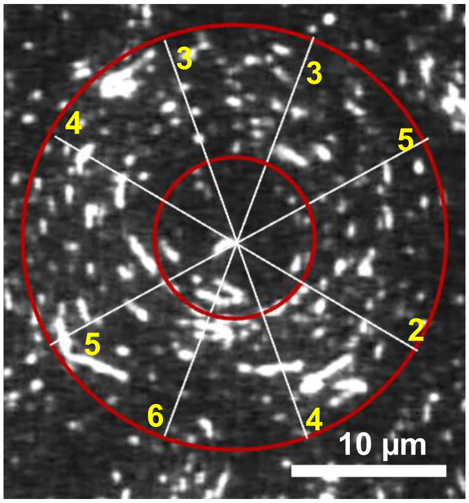

Fig. S8. Estimation of the number of actin filaments per ring width within the ACR.
To estimate the number of actin filaments spanning the ring width of an ACR, we used 24 nM of Cy3-labeled actin together with 2.4 µM of non-fluorescent actin, yielding a labeling ratio of 1:100. At a specific time point, four radial lines were drawn from the center of a representative ACR, generating eight radii. The number of Cy3-labeled actin filaments intersecting each radius within the defined ring width (i.e., the area between the inner and outer red circles) was counted. Each count was multiplied by 100 to estimate the total number of filaments, based on the labeling ratio. As shown in the figure, the yellow numbers represent the counts of labeled filaments per radius, with an average value of four. From this, the number of actin filaments within a ring width of 9 µm was estimated to be approximately 4 × 100 = 400. Assuming an actin filament diameter of 8 nm, the inter-filament spacing was estimated to be approximately 15 nm. Fig. 3D summarizes data from 20 ACRs, showing an average of 320 ± 100 filaments per ring width, with an inter-filament spacing of 22 ± 9.8 nm (mean ± standard deviation).

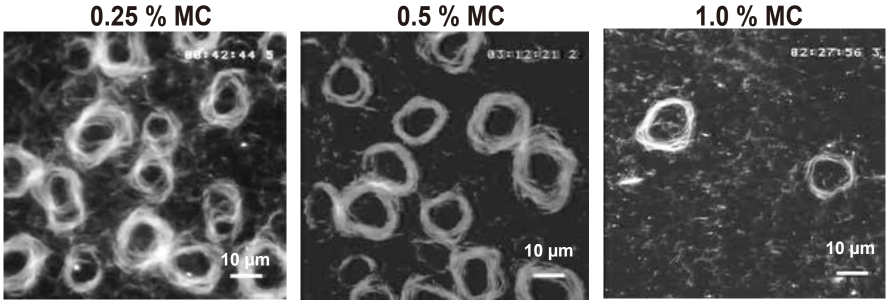

Fig. S9. ACRs formed in the presence of 0.25%, 0.5%, or 1.0% methylcellulose under the Bausch condition at an initial actin concentration of 2.4 µM (moderately high actin density). ACRs formed even in the presence of methylcellulose (MC) at viscosities comparable to that of bulk cytoplasm; however, the actin filaments within the rings appeared more densely packed. At 0.25% MC, ACRs were relatively stable. At 0.5% MC, some ACRs disappeared during observation. At 1.0% MC, instability was more pronounced, with frequent ACR disassembly. Representative time-lapse snapshots shown here are extracted from Movie S15. Experimental conditions are described in the *SI Appendix, Supplementary Materials and Methods, “in vitro* motility assay under the Bausch condition,” and summarized in Table S2. Scale bar, 10 µm.

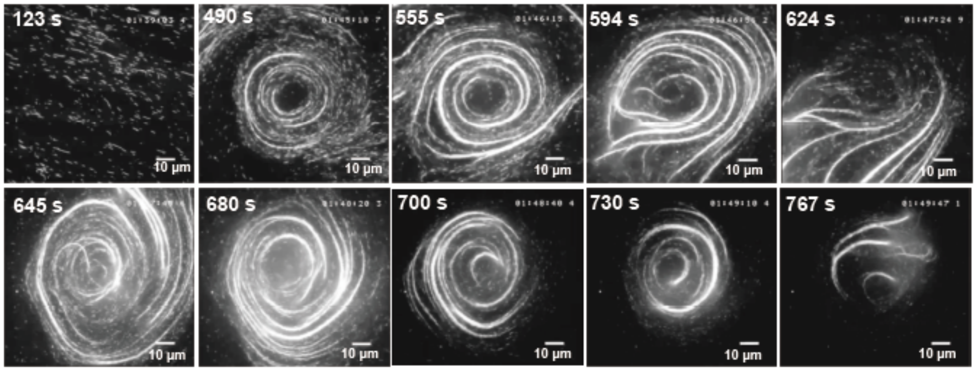

Fig. S10. Effect of methylcellulose on ACR formation under the Hatori condition. This figure shows the effect of 0.5% methylcellulose under the Hatori condition. Actin filaments (2.4 µM) were infused into a chamber coated with *Cc*XI MD, following the same loading protocol as the Bausch condition used for the moderately high–actin concentration. However, under the Hatori condition, the unbound-actin washing step was omitted to slightly increase the effective actin filament concentration near the surface, thereby enhancing methylcellulose-mediated depletion effects that promote actin filament bundling (10). Subsequently, an ATP solution containing 0.5% methylcellulose was added. Immediately after ATP perfusion, actin filaments exhibited nematic flow (123 s). Large CW-rotating vortices appeared several minutes after ATP perfusion. These vortices were unstable and disintegrated within 1–a few minutes after reaching their maximal size (490–624 s). At different fields of view within the same chamber, distinct vortices appeared and rapidly vanished (645–767 s), reflecting highly transient and unstable dynamics at this stage. Representative time-lapse snapshots shown here are extracted from Movie S16. Time 0 indicates the approximate moment of vortex formation rather than the onset of ATP perfusion. Experimental conditions are described in the *SI Appendix, Supplementary Materials and Methods, "in vitro* motility assay under the Hatori condition for methylcellulose assays," and summarized in Table S2.Scale bar, 10 µm.

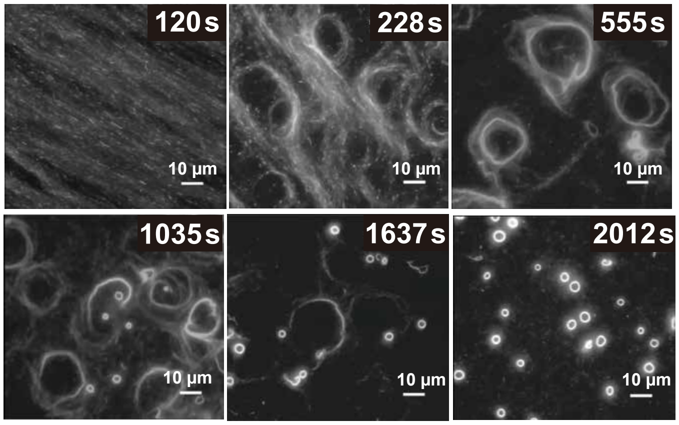

Fig. S11. Combined effects of villin and methylcellulose on ACR formation under the Hatori condition. This figure shows the combined effects of 1.6 µM villin and 0.5% methylcellulose under the Hatori condition. 2.4 µM actin filaments were infused into the chamber coated with *Cc*XI MD, the same protocol as Bausch condition under the moderately high-actin concentration. However, under the Hatori condition, the unbound-actin washing step was omitted to slightly increase the effective actin filament concentration near the surface, thereby enhancing methylcellulose-mediated depletion effects that promote actin filament bundling (10). Then, the ATP solution containing 0.5% methylcellulose was added. To characterize the temporal evolution of actin organization, representative time-lapse images capturing the most characteristic structures and dynamics at each time point are shown from six distinct fields of view within the same chamber (120 s, 228 s, 555 s, 1,035 s, 1,637 s, and 2,012 s after ATP addition). Immediately after ATP perfusion, actin filaments exhibited nematic flow. By ~200 s, large CW-rotating unstable ring-like structures with diameters of ~30 µm emerged. These numerous unstable large rings repeatedly formed and disassembled within several minutes at varying locations. At ~1,000 s, stable small ACRs (~5 µm), comparable to those formed with villin alone, appeared and persisted at fixed positions while exhibiting continuous CW rotation. By ~2,000 s, the unstable large ring-like structures had largely disappeared, and the chamber became predominantly occupied by stable small ACRs. Representative time-lapse snapshots shown here are extracted from Movie S17. Time indicates seconds after ATP perfusion. Experimental conditions are described in the *SI Appendix, Supplementary Materials and Methods, "in vitro* motility assay under the Hatori condition for methylcellulose assays," and summarized in Table S2.Scale bar, 10 µm.

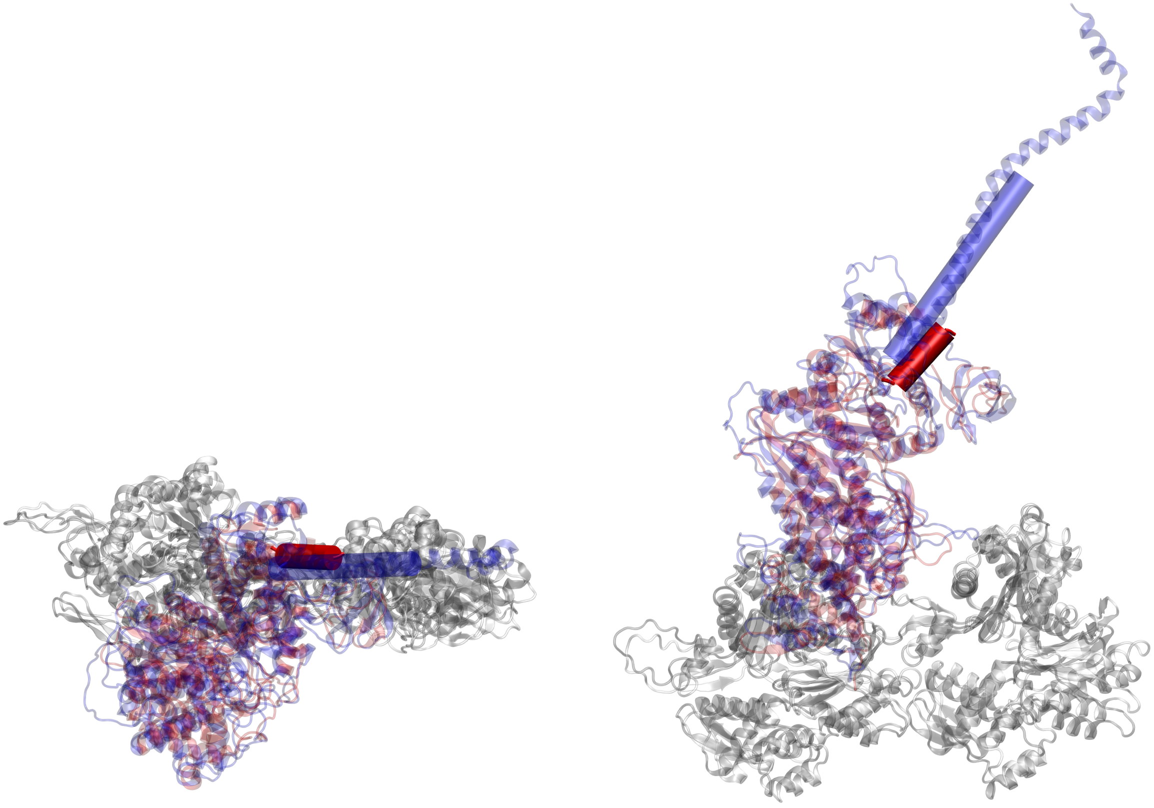

Top-down view   Side view

Fig. S12. Comparison of lever arm positions in acto-*Cc*XI MD and acto-SkII S1. The cryo-electron microscopy structures of acto-*Cc*XI MD (PDB ID: 7KCH, red) (23) and acto-SkII S1 (PDB ID: 5H53, blue) (24) were aligned at their motor domains to compare the positions of their lever arms. (Left) Top-down view along the filament axis, highlighting the relative positions of the lever arms after the power stroke. In this view, the lever arm position of acto-*Cc*XI MD in the rigor state appears nearly identical in orientation and position to that of acto-SkII S1, with both aligned almost parallel to the filament axis. (Right) Side view illustrating the spatial orientation of the lever arms after the power stroke. For clarity, the lever arms are depicted as cylinders, with the *Cc*XI lever arm shown as a red cylinder and the SkII lever arm as a blue cylinder.

Table S1. Summary of condition-dependent collective behaviors driven by *Cc*XI MD

Experimental condition	Initial actin conc.	Assay protocol*	Surface density of filaments† (filaments/μm²)	Dominant collective behaviors	ACR Formation	Figures /Movies
Bausch condition	2.4 μM	Actin loading → Wash unbound actin → ATP perfusion	15 ± 4.1 (n = 10) (moderately high actin density)	Transition from nematic streaming to robust ACR formation	Robust and frequent	Figs. 2B, 2C, 3A, 3B, 3D–3H, 4, Figs S3, S8, S9; Movies S2, S3, S7, and S11–S15
Bausch condition	7.1 μM	Actin loading → Wash unbound actin → ATP perfusion	41 ± 3.1 (n = 6) (high actin density)	Prolonged nematic streaming followed by polar streaming; delayed transition to ACR	Delayed and infrequent	Fig. S4; Movie S4
Bausch condition	24 μM	Actin loading → Wash unbound actin → ATP perfusion	75 ± 10 (n = 6) (ultra–high actin density)	Prolonged nematic streaming followed by persistent polar streaming; rare and spatially heterogeneous ACR formation	Rare and spatially heterogeneous	Fig. S5; Movie S5
Molloy condition	24 μM	Actin and ATP co-perfusion	71 ± 8.5 (n = 6) (ultra–high actin density)	Persistent nematic streaming	Not observed	Fig. S6 Movie S6

This table summarizes the dominant modes of collective actin filament motion observed under different experimental conditions in the *in vitro* motility assay driven by *Cc*XI MD. Initial actin concentration, assay protocol, estimated surface filament density, and ACR formation behavior are listed. ACRs formed robustly only within a moderately high filament density regime under the Bausch condition, whereas higher filament densities or the Molloy condition stabilized streaming states and suppressed ring formation.

*Details of each protocol are described in the *SI Appendix*, *Materials and Methods*.

† Surface filament densities were estimated as described in *SI Appendix*, Fig. S3.

Table S2. Summary of experimental conditions and outcomes for actin collective movements driven by CcXI MD in the presence of villin and/or methylcellulose (MC)

Experimental condition	Initial actin conc.	Assay protocol*	ACR Formation	Figures /Movies
Bausch condition + villin	2.4 µM	Actin loading → Wash unbound actin → ATP perfusion	Rapid formation of stable small ACRs	Fig. 4; Movie S14
Bausch condition + MC	2.4 µM	Actin loading → Wash unbound actin → ATP perfusion	Somewhat unstable ACR	Fig. S9; Movie S15
Hatori condition[1] + MC	2.4 µM	Actin loading → ATP perfusion with MC	Short-lived large vortices	Fig. S10; Movie S16
Hatori condition[1] + MC+ villin	2.4 µM	Actin loading → ATP perfusion with villin and MC	Eventual formation of stable small ACRs	Fig. S11 Movie S17

[1] Although the nominal actin concentration under the Hatori condition was 2.4 µM, identical to that under the Bausch condition, omission of the washing step resulted in a slight increase in the effective actin filament concentration near the surface. This enhanced methylcellulose-mediated depletion effects, promoting actin filament bundling (10).

*Details of each protocol are described in the *SI Appendix*, Materials and Methods.

Table S3. Predicted actin-binding residues of CcXI and SkII in the rigor state

Loops	*Cc*XI	Actin	SkII	Actin
CM loop (Cardiomyopathy loop)	A382<br>T389<br>K390<br>T383	A26<br>P333<br>E334<br>Y337	Y412<br>K415	A26, E334, Y337<br>P333
HTH (Helix-turn-helix)	M516<br>P518	L349<br>G146, R147	P529<br>M530<br>E538<br>M541<br>F542<br>K544<br>N552<br>K553	E4, Q353<br>S350, T351<br>L349, S350, T351<br>I345<br>Y143, V45<br>G146, T148<br>K50<br>T351
Loop 3	F544<br>K545	Y91, R95<br>R95	E99	K569

To identify the residues at the interfaces of *Cc*XI–actin (PDB ID: 7kcH) and SkII–actin (PDB ID: 5H53) in the rigor state, we used the PDBePISA server. Amino acids predicted by PDBePISA to participate in inter-chain interactions were defined as contact residues and compiled in Table S3. We selected PDBePISA rather than a simple distance-based cutoff (e.g., residues within 4.0 Å in PyMOL), because PDBePISA accounts for side-chain orientation and physicochemical context, thereby providing a more reliable estimate of potential binding interactions. While loop 2 and loop 4 were not resolved in the deposited 7kcH structure and thus could not be annotated, we documented the other actin-binding sites predicted from the models. Importantly, the amino acid residues involved in actin binding differ not only between *Cc*XI and SkII within each myosin binding loop, but also on the actin side, underscoring the divergence in actomyosin interfaces.

Movie S1-S17 (separate file)

Legends for Movies S1 to S17

Movie S1. Chiral curved motion of actin filaments driven by *Cc*XI MD This movie shows the chiral curved motion of actin filaments driven by *Cc*XI MD in the standard *in vitro* motility assay. Actin filaments moved clockwise (CW) when viewed from the objective lens side (myosin side). Experimental conditions are described in *SI Appendix*, *Supplementary Materials and Methods,* **"**Standard *in vitro* motility assay**"**. The movie is shown in real time. Scale bar, 10 μm.

Movie S2. Clockwise rotation of ACRs formed by *Cc*XI MD under the Bausch condition at an initial actin concentration of 2.4 μM (moderately high actin density). This movie shows the formation of actin chiral rings (ACRs) through collective filament motion under the Bausch condition at the initial actin concentration of 2.4 μM. ACRs emerged approximately 10 min after ATP addition and subsequently exhibited stable clockwise (CW) rotation at their formation sites. Experimental conditions are described in *SI Appendix, Supplementary Materials and Methods,* "*in vitro* motility assay under the Bausch condition" and summarized in Table S1. The movie is shown at 10× speed. Scale bar, 10 μm. (Related to Fig. 2B.)

Movie S3. Clockwise rotation of actin ACRs formed by *Cc*XI MD under the Bausch condition at the initial actin concentration of 2.4 μM (moderately high actin density) in 150 mM KCl. This movie shows CW-rotating ACRs formed under the Bausch condition at an initial actin concentration of 2.4 μM in 150 mM KCl (physiological ionic strength). ACRs formed and rotated stably, indicating that ACR formation is not restricted to the standard 25 mM KCl buffer. Experimental conditions are described in *SI Appendix, Supplementary Materials and Methods,***"***in vitro* motility assay under the Bausch condition**"**. The movie is shown at 10× speed. Scale bar, 10 μm.

Movie S4. Time-resolved transitions from nematic and polar streaming to ACR formation by *Cc*XI MD following ATP perfusion under the Bausch condition at the initial actin concentration of 7.1 μM (high actin density).

This movie illustrates the time-resolved collective dynamics of actin filaments after ATP perfusion in the i*n vitro* motility assay under the Bausch condition using an initial actin concentration of 7.1 μM unlabeled actin. Because the collective behaviors evolved over time and prolonged imaging at a single location caused significant photobleaching, each characteristic state was recorded at a different field of view within the same chamber, capturing representative dynamics at defined time points. Immediately after ATP addition, actin filaments exhibited nematic streaming, characterized by bidirectional alignment and flow (120–145 s). The orientation of this nematic streaming coincided with the direction of solution inflow into the chamber. As the system evolved, orientational symmetry gradually broke, and polar streaming emerged (480–550 s), characterized by unidirectional filament motion. At later time points, ACRs formed and stabilized (1,350–1,450 s). Compared with the moderately high– and high–actin density conditions (the Bausch condition at an initial actin concentration of 2.4 μM (Movie S7), corresponding to a filament density of 15 ± 4.1 filaments/μm², ACR formation required a longer time to occur, and the frequency of ACR appearance was notably lower under this higher actin density condition (41 ± 3.1 filaments/μm²). The nematic, polar, and ACR states shown here represent distinct collective regimes selected to visualize the temporal evolution of actin self-organization following ATP perfusion. Experimental conditions are described in *SI Appendix, Supplementary Materials and Methods*, "*in vitro* motility assay under the Bausch condition," and summarized in Table S1. The movie is shown at 10× speed. Scale bar, 10 μm. (Related to Fig. S4.)

Movie S5. Time-resolved transitions from nematic and polar streaming to ACR formation by *Cc*XI MD following ATP perfusion under the Bausch condition at the initial actin

concentration of 24 μM (ultra–high actin density). This movie illustrates the time-resolved collective dynamics of actin filaments after ATP perfusion in the in vitro motility assay under the Bausch condition using an initial concentration of 24 μM unlabeled actin. Because prolonged imaging at a single location caused photobleaching, each characteristic state was recorded from a different field of view within the same chamber. Immediately after ATP addition, actin filaments displayed nematic streaming (40–72 s), with the flow direction aligned with the direction of solution inflow. As time progressed, polar streaming became the dominant and persistent collective mode, appearing repeatedly across different locations (1,010–1,080 s and 2,190–2,250 s). ACRs first appeared at later time points (1,990–2,090 s). Notably, even after ACRs emerged, polar streaming persisted at other locations within the same chamber (e.g., 2,239 s), indicating that polar motion remained a prevalent collective state. At later times (2,360–2,400 s), ACRs were again observed at distinct positions, demonstrating that under this higher actin density condition, at which actin filaments were saturated at the surface, ACRs and persistent polar streaming coexisted spatially rather than converging into a single global pattern. Compared with the Bausch condition at an initial actin concentration of 2.4 μM (Movie S7) corresponding to a filament density of 15 ± 4.1 filaments/μm², this higher actin density condition (75 ± 10 filaments/μm²) exhibited a markedly prolonged predominance of polar streaming, resulting in delayed and infrequent ACR formation. The movie is shown at 10× speed. Scale bar, 10 μm. (Related to Fig. S5.)

Movie S6. Time-resolved persistent nematic streaming of actin filaments driven by *Cc*XI MD under the Molloy condition at the initial actin concentration of 24 μM. This movie shows time-lapse observations of actin filaments after perfusion of a solution containing 24 μM unlabeled actin filaments together with ATP into a chamber coated with CcXI MD (Molloy condition). To assess the reproducibility and spatial robustness of the collective dynamics, imaging was performed at four distinct fields of view within the same chamber. Throughout the ~1,600-s observation period, actin filaments aligned along their longitudinal axes and moved bidirectionally, a hallmark of nematic streaming, without forming higher-order structures such as actin chiral rings (ACR). Although the initial filament alignment reflected the direction of solution inflow, nematic streaming persisted even as local filament orientations varied across different regions of the chamber, confirming that this behavior reproducibly occurred at multiple locations on the same coverslip. Experimental conditions are described in the *SI Appendix, Supplementary Materials and Methods, "in vitro* motility assay under the Molloy condition," and summarized in Table S1. The movie is shown at 20× speed. Scale bar, 10 μm. (Related to Fig. S6.)

Movie S7. Continuous real-time process of ACR formation by *Cc*XI MD following ATP perfusion under the Bausch condition at the initial actin concentration of 2.4 μM (moderately high actin density). This movie captures the dynamic process of ACR formation following ATP addition: initial stream-like filament flow, coalescence into nascent rings, and maturation into stable ACRs, which rotated CW. Experimental conditions are described in *SI Appendix, Supplementary Materials and Methods*, "*in vitro* motility assay under the Bausch condition" and summarized in Table S1. The movie is shown at 40× speed, corresponding to 40–650 s after ATP addition. Scale bar, 10 μm. (Related to Fig. 2C.)

Movie S8. Simulation of collective motion of straight actin filaments. This movie illustrates a simulation of collective motion of straight actin filaments forming nematic streams. The filaments align along their longitudinal axes via nematic interactions, resulting in bidirectional streaming. The color represents the sine of the angle between each filament's movement direction and the x-axis (sin θ). Blue indicates motion with negative sin θ, and yellow indicates motion with positive sin θ. This behavior reflects the fundamental characteristics of nematic alignment in straight filament systems (see *SI Appendix*, *Supplementary Materials and Methods*, " Simulation on

motility assay of multiple actin filaments"). This movie is played at a rate of 15 s of simulation time per second of real time. Scale bar = 10 µm.

Movie S9. Simulation of collective motion of chiral curved actin filaments (persistence length = 10 µm), This movie illustrates a simulation of collective motion of chiral curved actin filaments, leading to the formation of ACRs (persistence length = 10 µm). This simulation corresponds to the control condition used for comparison in Movie S9. This control simulation replicates experimental observations and demonstrates that the collective motion of filaments with chiral curved motion results in the formation of ACRs (see *SI Appendix*, *Supplementary Materials and Methods,* " Simulation on motility assay of multiple actin filaments"). The color represents the sine of the angle between each filament's movement direction and the x-axis (sin θ); blue indicates negative sin θ and yellow indicates positive sin θ. This movie is played at a rate of 15 s of simulation time per second of real time. Scale bar = 10 µm. (Related to Fig. 2E.)

Movie S10. Simulations of collective motion of chiral curved filaments with different individual curvatures. This movie presents side-by-side simulations comparing the collective motion of chiral curved actin filaments with different degrees of single-filament curvature: left, low curvature; center, intermediate curvature; right, high curvature (see *SI Appendix, Supplementary Materials and Methods*, "Simulation on motility assay of multiple actin filaments"). In these simulations, the curvature of individual filaments is controlled by the persistence length, a parameter that determines single-filament curvature. Specifically, filament curvature was increased by reducing the persistence length (Lp): left, Lp = 10 µm (same simulation as Movie S8); center, Lp = 5 µm; right, Lp = 1 µm.
In all panels, filaments undergo chiral curved motion and spontaneously self-organize into rotating actin chiral rings (ACRs). As the curvature of individual filaments increases, the diameter of the resulting ACRs becomes progressively smaller.The color represents sin θ (blue, negative; yellow, positive). Playback rates are as follows: left and center panels, 15 s of simulation time per 1 s of real time; right panel, 1.5 s of simulation time per 1 s of real time. Scale bar, 10 µm. (Related to Fig. 3C.)

Movie S11. Fluorescence imaging of individual actin filaments within a fully formed ACR This movie shows trajectories of individual filaments within a fully formed ACR. Most filaments rotated CW, while ~10% rotated CCW. Filaments maintained stable, lane-preserving trajectories throughout the observation period. Compared with Movies S2–S6, a smaller amount of fluorescent actin was added during the observation phase, which reduced overall fluorescence but allowed clearer visualization of individual filament trajectories. Experimental conditions are described in *SI Appendix, Supplementary Materials and Methods*, "*in vitro* motility assay under the Bausch condition" and summarized in Table S1. The movie is shown at 2× speed. Scale bar, 10 µm. (Related to Fig. 3E.)

Movie S12. Collision of an actin filament with an ACR from the same direction as the ring's rotation This movie shows a filament colliding with an ACR from the same direction as the ring's CW rotation. The filament was incorporated and adopted CW rotation. Compared with Movies S2–S6, a smaller amount of fluorescent actin was added during the observation phase, which reduced overall fluorescence but allowed clearer visualization of individual filament trajectories. Experimental conditions are described in *SI Appendix, Supplementary Materials and Methods*, "*in*

vitro motility assay under the Bausch condition" and summarized in Table S1. The movie is shown in real time. Scale bar, 10 μm. (Related to Fig. 3G, upper panel.)

Movie S13. Collision of an actin filament with an ACR from the opposite direction to the ring's rotation This movie shows a filament colliding with an ACR from the opposite direction to its CW rotation. The filament was repelled without incorporation. Compared with Movies S2–S6, a smaller amount of fluorescent actin was added during the observation phase, which reduced overall fluorescence but allowed clearer visualization of individual filament trajectories. Experimental conditions are described in *SI Appendix, Supplementary Materials and Methods*, "*in vitro* motility assay under the Bausch condition" and summarized in Table S1. The movie is shown in real time. Scale bar, 10 μm. (Related to Fig. 3G, lower panel.)

Movie S14. Effect of villin on ACR formation. This movie shows the accelerated formation of small ACRs (~5 μm in outer diameter) driven by CcXI MD in the presence of 1.6 μM villin under the Bausch condition at an initial actin concentration of 2.4 μM. Within a few minutes after ATP perfusion, numerous small ACRs rapidly formed, whereas under the same condition without villin, ACR formation typically required ~10 min. Thus, villin accelerates ACR formation by several fold. Once formed, the small ACRs continued to rotate clockwise (CW) at fixed positions. A larger field of view than in other movies was recorded to capture more ACRs within a single frame. Experimental conditions are described in *SI Appendix, Supplementary Materials and Methods* ("*in vitro* motility assay under the Bausch condition") and summarized in Table S2. The movie is shown at 20× speed, corresponding to 40–290 s after ATP addition. Scale bar, 25 μm. (Related to Fig. 4.)

Movie S15. Stability of ACRs formed in the presence of increasing methylcellulose concentrations under the Bausch condition.

This movie compiles representative time-lapse recordings of actin chiral rings (ACRs) driven by *Cc*XI MD in the presence of 0.25%, 0.5%, or 1.0% methylcellulose (MC) under the Bausch condition at an initial actin concentration of 2.4 μM (moderately high actin density). At 0.25% MC, ACRs exhibited stable clockwise (CW) rotation. At 0.5% MC, some ACRs disassembled during observation, indicating reduced stability. At 1.0% MC, ACR formation was infrequent and instability was more pronounced, with frequent disassembly events. Representative time-lapse snapshots shown in Fig. S9 are extracted from this movie. Experimental conditions are described in *SI Appendix, Supplementary Materials and Methods,* "*in vitro* motility assay under the Bausch condition," and summarized in Table S2. The movie is shown at 10× speed. Scale bar, 10 μm. (Related to Fig. S9.)

Movie S16. Effect of methylcellulose on ACR formation under the Hatori condition. This movie shows the effect of 0.5% methylcellulose under the Hatori condition. Actin filaments (2.4 μM) were infused into a chamber coated with *Cc*XI MD, following the same loading protocol as the Bausch condition used for the moderately high–actin concentration. However, under the Hatori condition, the unbound-actin washing step was omitted to slightly increase the effective actin filament concentration near the surface, thereby enhancing methylcellulose-mediated depletion effects that promote actin filament bundling (10). Subsequently, an ATP solution containing 0.5% methylcellulose was added. To characterize the temporal evolution of actin organization, representative time-lapse images capturing the most characteristic structures and dynamics at each time point were recorded at four distinct fields of view within the same chamber (22–222 s, 490–630 s, 644–818 s, and 840–1115 s after ATP and methylcellulose addition). Immediately after the addition of ATP and methylcellulose, actin filaments exhibited nematic flow. Approximately 10 min after ATP and methylcellulose addition, large vortices (~100 μm) appeared. These vortices rotated clockwise (CW) but typically disassembled within a few minutes. Experimental conditions are described in the *SI Appendix, Supplementary Materials and Methods*, **"*in vitro* motility assay**

under the Hatori condition for methylcellulose assays," **and summarized in Table S2.** The movie is shown at 20× speed. Scale bar, 10 µm. (Related to Fig. S10.)

Movie S17. Combined effects of villin and methylcellulose on ACR formation under the Hatori condition This movie shows the combined effects of 1.6 µM villin and 0.5% methylcellulose under the Hatori condition. Actin filaments (2.4 µM) were infused into a chamber coated with *Cc*XI MD, following the same loading protocol as the Bausch condition used for the moderately high–actin concentration. However, under the Hatori condition, the unbound-actin washing step was omitted to slightly increase the effective actin filament concentration near the surface, thereby enhancing methylcellulose-mediated depletion effects that promote actin filament bundling (10). Subsequently, an ATP solution containing 1.6 µM villin and 0.5% methylcellulose was added. To characterize the temporal evolution of actin organization, representative time-lapse images capturing the most characteristic structures and dynamics at each time point were recorded at four distinct fields of view within the same chamber (60–95 s, 565–603 s, 1,605–1,645 s, and 2,014–2,074 s after ATP addition).Immediately after ATP, villin, and methylcellulose addition, actin filaments exhibited nematic flow, which gradually transitioned into unstable large rings that typically disassembled by ~1,000 s. Subsequently, stable small ACRs (~5 µm in diameter), comparable to those formed in the presence of villin alone (Fig. S8 and Movie S14), began to appear. Experimental conditions are described in the *SI Appendix, Supplementary Materials and Methods,* "*in vitro* motility assay under the Hatori condition for methylcellulose assays," and summarized in Table S2. The movie is shown at 5× speed. Scale bar, 10 µm. (Related to Fig. S11.)

SI References

1. T. Haraguchi *et al.*, Discovery of ultrafast myosin, its amino acid sequence, and structural features. *Proceedings of the National Academy of Sciences* **119**, e2120962119 (2022).
2. K. Ito *et al.*, Recombinant motor domain constructs of Chara corallina myosin display fast motility and high ATPase activity. *Biochemical and Biophysical Research Communications* **312**, 958-964 (2003).
3. K. Ito *et al.*, Kinetic mechanism of the fastest motor protein, Chara myosin. *J Biol Chem* **282**, 19534-19545 (2007).
4. K. Ito, Y. Yamaguchi, K. Yanase, Y. Ichikawa, K. Yamamoto, Unique charge distribution in surface loops confers high velocity on the fast motor protein <i>Chara</i> myosin. *Proceedings of the National Academy of Sciences* **106**, 21585-21590 (2009).
5. V. Schaller, C. Weber, C. Semmrich, E. Frey, A. R. Bausch, Polar patterns of driven filaments. *Nature* **467**, 73-77 (2010).
6. M. Tominaga *et al.*, Cytoplasmic streaming velocity as a plant size determinant. *Dev Cell* **27**, 345-352 (2013).
7. T. Haraguchi *et al.*, Functional Diversity of Class XI Myosins in Arabidopsis thaliana. *Plant Cell Physiol* **59**, 2268-2277 (2018).
8. S. Rula *et al.*, Measurement of enzymatic and motile activities of Arabidopsis myosins by using Arabidopsis actins. *Biochemical and Biophysical Research Communications* **495**, 2145-2151 (2018).
9. T. Butt *et al.*, Myosin motors drive long range alignment of actin filaments. *J Biol Chem* **285**, 4964-4974 (2010).
10. T. Iwase, Y. Sasaki, K. Hatori, Alignment of actin filament streams driven by myosin motors in crowded environments. *Biochimica et Biophysica Acta (BBA) - General Subjects* **1861**, 2717-2725 (2017).
11. M. van Ginkel, J. Weijer, L. Van Vliet, P. Verbeek, *Curvature Estimation from Orientation Fields* (1999).
12. K. Shiba, Y. Mogami, S. Baba, Ciliary Movement of Sea-urchin Embryos. *Nat Sci Rep Ochanomizu Univ* **53** (2001).
13. T. Nitta, A. Tanahashi, M. Hirano, In silico design and testing of guiding tracks for molecular shuttles powered by kinesin motors. *Lab on a Chip* **10**, 1447-1453 (2010).
14. G. Skačej, V. M. Pergamenshchik, A. L. Alexe-Ionescu, G. Barbero, S. Žumer, Subsurface deformations in nematic liquid crystals: The hexagonal lattice approach. *Physical Review E* **56**, 571-580 (1997).
15. T. Kalwarczyk *et al.*, Comparative Analysis of Viscosity of Complex Liquids and Cytoplasm of Mammalian Cells at the Nanoscale. *Nano Letters* **11**, 2157-2163 (2011).
16. M. Weiss, M. Elsner, F. Kartberg, T. Nilsson, Anomalous Subdiffusion Is a Measure for Cytoplasmic Crowding in Living Cells. *Biophysical Journal* **87**, 3518-3524 (2004).
17. K. Fushimi, A. S. Verkman, Low viscosity in the aqueous domain of cell cytoplasm measured by picosecond polarization microfluorimetry. *J Cell Biol* **112**, 719-725 (1991).
18. B. Büyükurgancı *et al.*, Shear rheology of methyl cellulose based solutions for cell mechanical measurements at high shear rates. *Soft Matter* **19**, 1739-1748 (2023).
19. R. Moreira, F. Chenlo, C. Silva, M. D. Torres, Rheological behaviour of aqueous methylcellulose systems: Effect of concentration, temperature and presence of tragacanth. *LWT* **84**, 764-770 (2017).
20. S. Köhler, O. Lieleg, A. R. Bausch, Rheological Characterization of the Bundling Transition in F-Actin Solutions Induced by Methylcellulose. *PLOS ONE* **3**, e2736 (2008).
21. M. Miyazaki, M. Chiba, H. Eguchi, T. Ohki, S. i. Ishiwata, Cell-sized spherical confinement induces the spontaneous formation of contractile actomyosin rings in vitro. *Nature Cell Biology* **17**, 480-489 (2015).
22. D. Popp, A. Yamamoto, M. Iwasa, Y. Maéda, Direct visualization of actin nematic network formation and dynamics. *Biochemical and Biophysical Research Communications* **351**, 348-353 (2006).
23. P. V. Ruijgrok *et al.*, Optical control of fast and processive engineered myosins in vitro and in living cells. *Nature Chemical Biology* **17**, 540-548 (2021).

24. T. Fujii, K. Namba, Structure of actomyosin rigour complex at 5.2 A resolution and insights into the ATPase cycle mechanism. *Nat Commun* **8**, 13969 (2017).
